# Quaternary Selenides EuLnCuSe_3_: Synthesis, Structures, Properties and In Silico Studies

**DOI:** 10.3390/ijms23031503

**Published:** 2022-01-28

**Authors:** Maxim V. Grigoriev, Leonid A. Solovyov, Anna V. Ruseikina, Aleksandr S. Aleksandrovsky, Vladimir A. Chernyshev, Dmitriy A. Velikanov, Alexander A. Garmonov, Maxim S. Molokeev, Aleksandr S. Oreshonkov, Nikolay P. Shestakov, Alexey V. Matigorov, Svetlana S. Volkova, Evgeniy A. Ostapchuk, Alexander V. Kertman, Thomas Schleid, Damir A. Safin

**Affiliations:** 1 Laboratory of Theory and Optimization of Chemical and Technological Processes, University of Tyumen, 625003 Tyumen, Russia; maxgrigmvv@yandex.ru (M.V.G.); ostapchuk_evgeniy@list.ru (E.A.O.); 2 Institute of Chemistry and Chemical Technology, Federal Research Center KSC SB RAS, 660036 Krasnoyarsk, Russia; leosol@icct.ru; 3 Institute of Chemistry, University of Tyumen, 625003 Tyumen, Russia; s.s.volkova@utmn.ru (S.S.V.); a.v.kertman@utmn.ru (A.V.K.); 4 Department of Photonics and Laser Technology, Siberian Federal University, 660079 Krasnoyarsk, Russia; aleksandrovsky@kirensky.ru; 5 Kirensky Institute of Physics, Federal Research Center KSC SB RAS, 660036 Krasnoyarsk, Russia; dpona1@gmail.com (D.A.V.); msmolokeev@mail.ru (M.S.M.); oreshonkov@iph.krasn.ru (A.S.O.); nico@iph.krasn.ru (N.P.S.); 6 Institute of Natural Sciences and Mathematics, Ural Federal University Named after the First President of Russia B.N. Yeltsin, Mira Str. 19, 620002 Ekaterinburg, Russia; vchern@inbox.ru; 7 Institute of Physics and Technology, University of Tyumen, 625003 Tyumen, Russia; gamma125@mail.ru; 8 Research and Development Department, Kemerovo State University, 650000 Kemerovo, Russia; 9 Institute of Engineering Physics and Radioelectronic, Siberian Federal University, 660079 Krasnoyarsk, Russia; 10 School of Engineering and Construction, Siberian Federal University, 660041 Krasnoyarsk, Russia; 11 Engineering Centre of Composite Materials Based on Wolfram Compounds and Rare-Earth Elements, University of Tyumen, 625003 Tyumen, Russia; a.v.matigorov@utmn.ru; 12 Institute of Inorganic Chemistry, University of Stuttgart, D-70569 Stuttgart, Germany; thomas.schleid@iac.uni-stuttgart.de; 13 Advanced Materials for Industry and Biomedicine Laboratory, Kurgan State University, Sovetskaya Str. 63/4, 640020 Kurgan, Russia; 14 Innovation Center for Chemical and Pharmaceutical Technologies, Ural Federal University Named after the First President of Russia B.N. Yeltsin, Mira Str. 19, 620002 Ekaterinburg, Russia

**Keywords:** inorganic materials, ab initio calculations, magnetic measurements, lattice dynamics, vibrational spectroscopy, optical spectroscopy, negative magnetization

## Abstract

In this work, we report on the synthesis, in-depth crystal structure studies as well as optical and magnetic properties of newly synthesized heterometallic quaternary selenides of the Eu^+2^Ln^+3^Cu^+1^Se_3_ composition. Crystal structures of the obtained compounds were refined by the derivative difference minimization (DDM) method from the powder X-ray diffraction data. The structures are found to belong to orthorhombic space groups *Pnma* (structure type Ba_2_MnS_3_ for EuLaCuSe_3_ and structure type Eu_2_CuS_3_ for EuLnCuSe_3_, where Ln = Sm, Gd, Tb, Dy, Ho and Y) and *Cmcm* (structure type KZrCuS_3_ for EuLnCuSe_3_, where Ln = Tm, Yb and Lu). Space groups *Pnma* and *Cmcm* were delimited based on the tolerance factor t’, and vibrational spectroscopy additionally confirmed the formation of three structural types. With a decrease in the ionic radius of Ln^3+^ in the reported structures, the distortion of the (LnCuSe_3_) layers decreases, and a gradual formation of the more symmetric structure occurs in the sequence Ba_2_MnS_3_ → Eu_2_CuS_3_ → KZrCuS_3_. According to magnetic studies, compounds EuLnCuSe_3_ (Ln = Tb, Dy, Ho and Tm) each exhibit ferrimagnetic properties with transition temperatures ranging from 4.7 to 6.3 K. A negative magnetization effect is observed for compound EuHoCuSe_3_ at temperatures below 4.8 K. The magnetic properties of the discussed selenides and isostructural sulfides were compared. The direct optical band gaps for EuLnCuSe_3_, subtracted from the corresponding diffuse reflectance spectra, were found to be 1.87–2.09 eV. Deviation between experimental and calculated band gaps is ascribed to lower *d* states of Eu^2+^ in the crystal field of EuLnCuSe_3_, while anomalous narrowing of the band gap of EuYbCuSe_3_ is explained by the low-lying charge-transfer state. Ab initio calculations of the crystal structures, elastic properties and phonon spectra of the reported compounds were performed.

## 1. Introduction

Nowadays, the copper-containing chalcogenides are of particular interest and in the limelight of many studies, the reason being their thermal, electrical and optical properties, which make them promising materials for infrared (IR) and nonlinear optics [1,2], photocatalysis, photoelectric energy and proton conductivity [3,4,5,6,7,8,9,10,11,12,13,14,15,16,17,18]. Development of electronic technologies opened prospects for the copper containing selenides as one of the most promising absorbers in thin film photovoltaic cells and high-performance thermoelectric materials [19,20]. Copper selenides can be used as bifunctional electrocatalysts for water splitting in an alkaline medium with low overvoltages for the oxygen evolution reaction (OER) at the anode and hydrogen evolution reaction (HER) at the cathode [21].

Quaternary selenides, comprising copper and rare earth elements, are of particular interest due to a variety of different combinations of cations that enable the design of the structural type and band gap, as well as electrical and optical characteristics [1,2,3,8,22,23,24,25,26,27,28,29,30,31,32,33]. Recent theoretical studies well predict thermoelectric properties of the copper-derived quaternary selenides [32,34,35], which are one of the promising alternatives to traditional thermoelectric materials. Notably, copper-containing chalcogenides are more abundant in the Earth’s crust and are relatively cheap in comparison to the bismuth- and lead-derivatives [36].

Selenides of the type A^+2^Ln^+3^Cu^+1^Se^−2^_3_ (A = Sr, Ba, Pb) crystallize in several orthorhombic structural types, namely Ba_2_MnS_3_ (space group *Pnma*), BaLaCuS_3_ (space group *Pnma*), Eu_2_CuS_3_ (space group *Pnma*), KZrCuS_3_ (space group *Cmcm*) (Table S1 in the Supplementary Materials) [1,3,25,26,27,28,30,36,37,38,39,40,41,42,43]. Additionally, the magnetic and optical properties of these compounds have also been described [1,3,8,30]. Quaternary selenides have a high energy conversion efficiency with a figure of merit higher than unity at 600 K [32]. Due to a 4f–5d transition in the Eu^2+^ ion, incorporation of this ion into the compound yields in a narrower band gap [9], than, e.g., 1.96 eV obtained for BaGdCuSe_3_ [3]. For a series of thermodynamically stable quaternary chalcogenides ALnCuSe_3_ (A = Sr, Ba, Pb, Eu), quantum mechanical calculations revealed a very low thermal conductivity of the lattice due to its strong anharmonicity, which enhances phonon scattering [32]. Compounds with low thermal conductivity can be used as high-performance thermoelectrics in thermal barrier coatings and thermal data-storage devices [32,44,45,46,47].

Selenides of the rhombic system EuLnCuSe_3_ (Ln = La–Lu, Sc, Y) with the band gap of 0.82–1.09 eV were also predicted [32]. Quaternary selenides EuLnCuSe_3_ are also indirectly predicted by the existence of tertiary selenides CuLnSe_2_ [18,48,49]. The former compounds should form in the CuLnSe_2_–EuS section of the EuS–Ln_2_Se_3_–Cu_2_S ternary system analogously to the chalcogenides of a similar composition [38,39,40,41,50,51,52,53,54,55,56,57,58,59]. Three structural types were predicted for EuLnCuSe_3_, namely KZrCuSe_3_ (Ln = Nd–Lu, Y), BaLaCuS_3_ (Ln = La–Pr) and NaCuTiS_3_ for EuScCuSe_3_ [32]. This contradicts the data obtained for the synthesized Eu^+2^Eu^+3^Cu^+1^Se^−2^_3_, for which the structural type of Eu_2_CuS_3_ with the cell parameters *a* = 10.773(7) Å, *b* = 4.134(3) Å, *c* = 13.466(9) Å was established [31]. This compound was synthesized from elemental europium and selenium for about 400 h [31]. The resulting sample was contaminated by EuSe and an unidentified impurity. It should also be noted that selenides EuLnCuSe_3_ (Ln = Nd, Sm, Gd, Er) were obtained very recently [60]. However, the applied synthetic procedure was remarkably time-consuming and allowed the production of only significantly contaminated samples. Furthermore, only EuErCuSe_3_ was structurally characterized, and its thermal, optical and magnetic properties were studied.

Finally, a similarity in the structural types of EuLnCuSe_3_ and SrLnCuSe_3_ could be observed, since the radius of Eu^2+^ (1.17 Å, coordination number = 6 [61]) is close to the radius of Sr^2+^ (1.18 Å, coordination number = 6 [61]), which was observed for the sulfides EuLnCuS_3_ [10] and SrLnCuS_3_ [51]. For a series of selenides SrLnCuSe_3_ (Ln = La–Lu, Y, Sc), the existence of three structural types Ba_2_MnS_3_, Eu_2_CuS_3_ and KZrCuS_3_ (Table S1 in the Supplementary Materials) was experimentally established [26,27,28,36,42]. Thus, we expect similar structural types for the EuLnCuSe_3_ selenides.

With all this in mind, in this work we directed our attention towards a series of quaternary selenides EuLnCuSe_3_ (Ln = La, Sm, Gd, Tb, Dy, Ho, Y, Tm, Lu). Particularly, we focused on the efficient synthetic approach, crystal structure determination, optical and magnetic properties as well as computational analysis of the obtained structures. All the obtained results were examined with respect to the Ln^3+^ radius.

## 2. Results and Discussion

### 2.1. Synthesis of Selenides

Quaternary selenides reported in this work were obtained from the corresponding starting materials, which were carefully prepared before the synthesis by thermolysis of the corresponding co-crystallized metal nitrates. Both Eu_2_O_3_ and copper were pretreated as described in the literature [9]. Oxides of rare earth elements were annealed 1070 K to remove sorption water, hydroxides, hydrocarbonates and oxycarbonates [62,63], while Tb_4_O_7_ was annealed at 470 K to keep its chemical composition and avoid a stepwise transformation: Tb_4_O_7_ → Tb_11_O_20_ → Tb_7_O_12_ → Tb_2_O_3_ [64]. The completeness of thermolysis was proved by the elimination of absorption bands in the corresponding IR spectra due to vibrations of the nitrate and hydroxyl groups. The absence of nitrogen-containing compounds was also confirmed by the distribution spectra of elements, plotted using an energy dispersive analysis system, in which no characteristic lines of nitrogen were observed, while the presence of characteristic lines of four elements O, Ln, Eu, Cu indicated the formation of oxides. According to X-ray phase analysis, the resulting samples comprised Cu_2_Ln_2_O_5_, CuO, (Ln/Eu)_2_O_3_, (Eu/Ln)_2_CuO_4_. Notably, the selenidation of complex oxides decreases the synthesis time of EuLnCuSe_3_ in comparison with the selenidation of a mixture of commercial oxides CuO, Ln_2_O_3_, Eu_2_O_3_. Finally, compounds EuLnCuSe_3_ in the powdered form were prepared by the reductive selenidation of the oxide mixtures, yielding aggregated particles with linear sizes up to about 30 μm (Figure S1 in the Supplementary Materials). The energy dispersive X-ray spectrometric analysis data are in agreement with the calculated data (see the Section 3.2 *Synthesis*). According to the X-ray phase analysis, the content of the main phase in the samples ranges from 95.7% to 100% (Figure S2 in the Supplementary Materials). Notably, the recently reported synthetic approach allowed the isolation of selenides with the main phase content of only 87.4–96.0% [60]. Furthermore, the described herein synthetic procedure significantly decreases the reaction time down to about 24 h at the temperature 970–1170 K.

### 2.2. Crystal Structures

**Figure 1:**
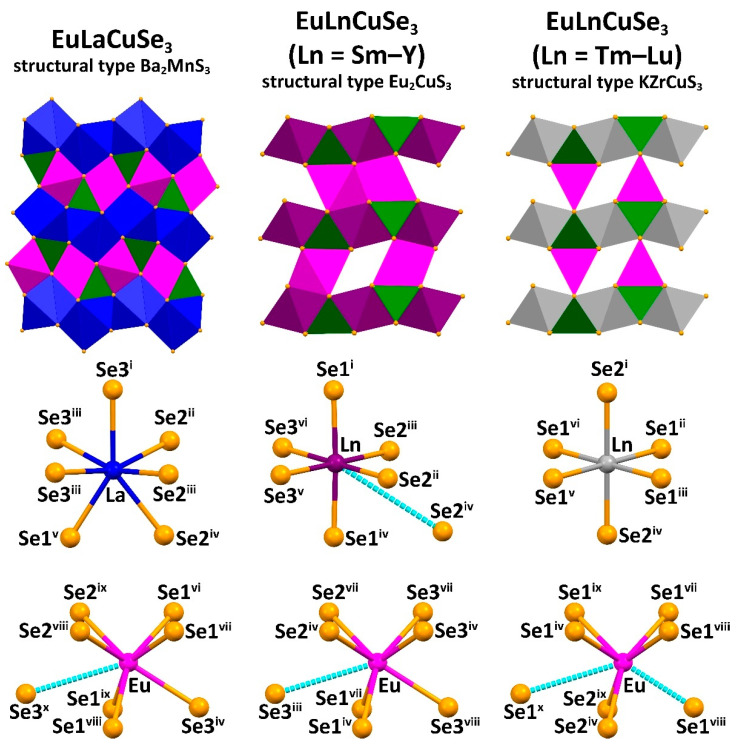
Best views of the crystal structures of EuLaCuSe_3_, EuLnCuSe_3_ (Ln = Sm–Y) and EuLnCuSe_3_ (Ln = Tm–Lu) (top row). Coordination environments around the Ln^3+^ (middle row) and Eu^2+^ (bottom row) cations in the structures of the reported selenides. For bond distances, see Table S4 in the Supplementary Materials. Color code: magenta polyhedron = EuSe_7_ for EuLnCuSe_3_ (Ln = La–Y) and EuS_6_ for EuLnCuSe_3_ (Ln = Tm–Lu), green polyhedron = CuS_4_, blue polyhedron = LaSe_7_, purple polyhedron = LnSe_6_ (Ln = Sm–Y) and grey polyhedron = LnSe_6_ (Ln = Tm–Lu).

**Figure 2:**
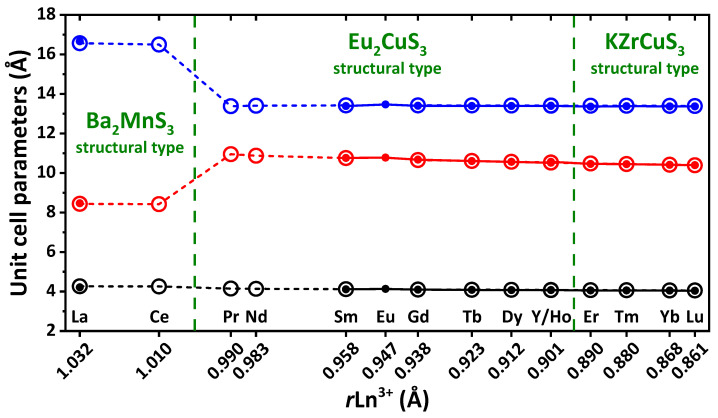
The calculated (open circles) and experimental (filled circles) unit cell parameters in the structures of EuLnCuSe_3_ (Ln = La, Sm, Eu [31], Gd, Tb, Dy, Ho, Y, Er [60], Tm, Yb, Lu). Black = *a* and *b* axes for space groups *Cmcm* and *Pnma*, respectively; red = *c* and *a* axes for space groups *Cmcm* and *Pnma*, respectively; blue = *b* and *c* axes for space groups *Cmcm* and *Pnma*, respectively.

The crystal structures of EuLnCuSe_3_ were best solved in the orthorhombic system from powder X-ray diffraction patterns (Figure 1, Table 1, Figure S2 and Tables S2–S5 in the Supplementary Materials). The calculated unit cell parameters obtained using the PBE0 density functional theory (DFT) functional are in good agreement with the experimental values (Figure 2, Table 1 and Table S6 in the Supplementary Materials). Notably, the PBE0 functional was recently shown to be efficient to reproduce crystal structures, phonon spectra and elastic properties of compounds with ionic and covalent bonds [65]. Furthermore, it was also shown that the use of pseudopotentials for rare-earth ions replacing the inner shells, including 4*f* orbitals, allows the successful description of the structure and dynamics of the lattice of compounds with a sublattice of rare-earth ions [66].

Two types of orthorhombic *Pnma* and one type of orthorhombic *Cmcm* crystal structures were revealed for EuLnCuSe_3_ (Figure 1 and Figure 2). Compound EuLaCuSe_3_ belongs to the structural type Ba_2_MnS_3_, while compounds EuLnCuSe_3_ (Ln = Sm, Eu [31], Gd, Tb, Dy, Ho, Y) are isostructural to Eu_2_CuS_3_. These crystal structures have the same type of symmetry but differ in the system of bonds and coordination of Ln^3+^. Finally, selenides EuLnCuSe_3_ (Ln = Tm, Yb, Lu) belong to the structural type KZrCuS_3_. In all compounds, the Eu^2+^ and Ln^3+^ cations each occupy two crystallographically independent positions.

The distorted tetrahedra CuSe_4_ are linked by vertex atoms yielding polymeric chains along the *b* axis for the structural types Ba_2_MnS_3_ and Eu_2_CuS_3_, and along the *a* axis for the structural type KZrCuS_3_. In the structure of EuLaCuSe_3_, the polyhedron EuSe_7_ is formed from two atoms of Se1^i^, two atoms of Se1^ii^, two atoms of Se2^i^ and one atom of Se3 (Table S4 in the Supplementary Materials) with all the Eu–Se distances being within the calculated sum of ionic radii 3.15–3.18 Å (*r*Se^2−^ = 1.98 Å (coordination number = 6); *r*Eu^2+^ = 1.17 Å (coordination number = 6); *r*Eu^2+^ = 1.20 Å (coordination number = 7) [61]). The eighth selenide ion is located at a distance that significantly exceeds the sum of ionic radii (>4 Å). The layered structures of EuLnCuSe_3_ (Ln = Eu–Lu) are formed by two-dimensional layers [LnCuSe_3_] in the *ab* plane for EuLnCuSe_3_ (Ln = Eu–Y) and in the *ac* plane for EuLnCuSe_3_ (Ln = Tm–Lu), between which the Eu^2+^ ions are located.

In the EuLnCuSe_3_, as the radius *r*Ln^3+^ decreases, the average Ln–Se distance gradually decreases from 3.09(7) Å down to 2.82(1) Å, while the Cu–Se distance remains intact and of 2.46(2)–2.48(3) Å (Table S4 in the Supplementary Materials). Notably, since the distorted octahedra LnSe_6_ are linked into layers with the CuSe_4_ tetrahedra, as *r*Ln^3+^ decreases, the crystal-chemical compression of these layers is observed. A decrease in the coordination saturation of the lanthanide cation leads to changes in the coordination polyhedron, structural type and space group (Figure 1). Particularly, changing the coordination polyhedron from the monocapped trigonal prism LnSe_7_ in EuLaCuSe_3_ to the LnSe_6_ octahedron (Ln = Eu–Y) leads to the change in the structural type Ba_2_MnS_3_ to Eu_2_CuS_3_, and the monocapped trigonal prism EuSe_7_ in EuLnCuSe_3_ (Ln = Eu–Y) to the trigonal prism EuSe_6_ in EuLnCuSe_3_ (Ln = Tm–Lu) leads to the change in the structural type Eu_2_CuS_3_ to KZrCuS_3_. A similar change in the structural types was observed for SrLnCuSe_3_ (Ln = La–Lu) [26,27,28,36,42] and EuLnCuS_3_ [10]. Thus, the change in the structural type in EuLnCuSe_3_ is dictated by the size of the Ln^3+^ cation.

**Figure 3:**
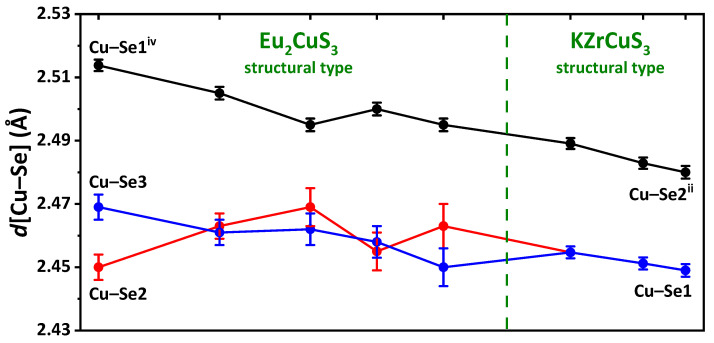

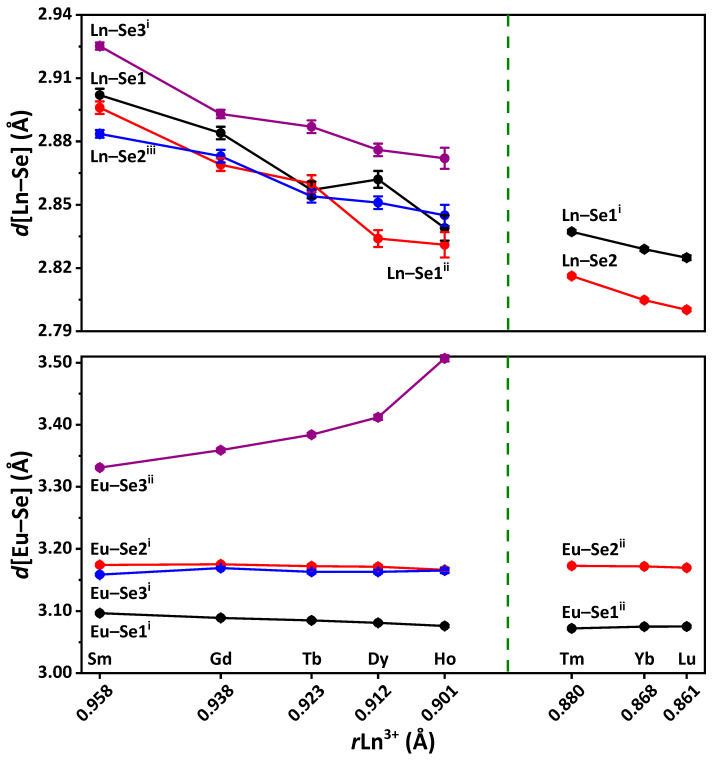
The M–Se distances in the structures of EuLnCuSe_3_ (Ln = Sm, Gd, Tb, Dy, Ho, Tm, Yb, Lu).

In the structures of EuLnCuSe_3_ (Ln = Sm, Gd, Tb, Dy, Ho, Y; structural type Eu_2_CuS_3_), six Eu–Se distances, namely two Eu–Se1^i^, two Eu–Se2^i^ and two Eu–Se3^i^, are shorter than 3.2 Å, while the seventh and the longest distance Eu–Se3^ii^ gradually increases in the following sequence: 3.331 Å (EuSmCuSe_3_) → 3.359 Å (EuGdCuSe_3_) → 3.384 Å (EuTbCuSe_3_) → 3.412 Å (EuDyCuSe_3_) → 3.424 Å (EuYCuSe_3_) → 3.507 Å (EuHoCuSe_3_) (Figure 3, Table S4 in the Supplementary Materials). A similar trend was observed for isostructural compounds EuLnCuS_3_ [10] and SrLnCuS_3_ [67].

In the structures of EuLnCuSe_3_ (Ln = Tm, Yb, Lu; structural type KZrCuS_3_), six Eu–Se distances, namely four Eu–Se1^ii^ and two Eu–Se2^ii^, are also shorter than 3.2 Å, while two more separations Eu–Se1 are longer than 3.55 Å and, thus, are not included in the coordination sphere of the metal ion (Figure 1, Table S4 in the Supplementary Materials).

In compounds EuLnCuSe_3_ (Ln = La–Lu), the unit cell parameters *b* (space group *Pnma*) and *a* (space group *Cmcm*) decrease. The parameters *a* (space group *Pnma*) and *c* (space group *Cmcm*) also decrease within each structural type of EuLnCuSe_3_ (Ln = Sm–Ho) and EuLnCuSe_3_ (Ln = Tm–Lu). The unit cell parameters *a* and *c* (space group *Pnma*) upon the changing of the structural type from Ba_2_MnS_3_ to Eu_2_CuS_3_ abruptly increase and decrease, respectively. The unit cell volume changes from EuHoCuSe_3_ to EuTmCuSe_3_ smoothly, despite the change in symmetry. A similar process was observed for isomorphic compounds ALnMX_3_ (A = Sr, Eu, Ba; M = Cu, Ag; X = S, Se, Te) [25,29,68]. All these changes clearly testify to morphotropy in a series of the reported EuLnCuSe_3_ compounds.

Despite that EuYCuSe_3_ belongs to the same structural type as EuLnCuSe_3_ (Ln = Sm–Ho), and the yttrium ion radius is less than that of holmium, the unit cell parameters of the yttrium-based selenide are larger than those for EuDyCuSe_3_ and EuHoCuSe_3_, which is in agreement with the data obtained for isostructural quaternary sulfides ALnCuS_3_ (A = Sr, Eu) [10,42,68,69]. This can be explained by the electronic structure of yttrium, which is a *d*-element.

**Figure 4:**
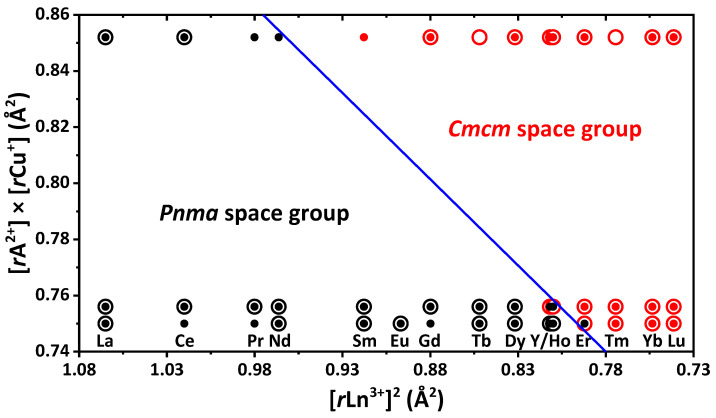
Discrimination between space groups for the structures of ALnCuS_3_ (filled circles) and ALnCuSe_3_ (open circles) depending on the ionic radii of the metal ions. A = Ba (top row), Sr (middle row) and Eu (bottom row).

In general, the EuLnCuSe_3_ compounds are isostructural to EuLnCuS_3_ [10]. When selenium is included instead of sulfur, the anionic sublattice expands, since selenium is a larger anion. A comparison of sulfide and selenide analogues EuLnCuX_3_ (X = S, Se, Ln = La–Lu, Y) allows the reveal of the formation of different types of crystal structures with the same rare-earth metals (Figure 4). Particularly, SrYCuS_3_ and SrHoCuS_3_ crystallize in space group *Pnma*, while their selenide derivatives crystallize in space group *Cmcm* (Figure 4) [28,42,53,69].

In the structures of SrYCuSe_3_ and EuYCuSe_3_, the Sr^2+^ and Eu^2+^ ions exhibit different coordination polyhedra. Compound SrYCuSe_3_ contains the SrSe_6_ polyhedron with the most pronounced ionic character of the included atoms, having a local symmetry of 43*m* (highly symmetric coordination), while EuYCuSe_3_ contains the EuSe_7_ polyhedron with a less pronounced ionic character of atoms, with the *mm*2 symmetry (less symmetric coordination). As the ionic radius of the lanthanide *r*Ln^3+^ decreases in ALnCuSe_3_, the formation of a highly symmetric coordination of A^2+^ occurs earlier in SrLnCuSe_3_ than in EuLnCuSe_3_. Particularly, SrSe_7_ (*Pnma*) transforms into SrSe_6_ (*Cmcm*) from Dy to Ho [42], while the change in the coordination polyhedron of europium occurs later, viz. from Y to Er. A similar process was an observed trend for ALnCuS_3_ (A = Eu, Sr) [10,42]. In SrLnCuS_3_ and SrLnCuS_3_, for example, the change in the strontium and europium coordination polyhedra is observed from Y to Er and from Er to Tm, respectively [10,42]. This is due to the more pronounced ionic characteristics of the alkaline earth elements in comparison with the rare earth metals.

Thus, with an increase in the degree of ionicity of the A^2+^–X bond, as well as the A^2+^ radius, the number of compounds, crystallized in the *Cmcm* space group, increases, and the A^2+^ ions become six-coordinated earlier.

To separate orthorhombic space groups *Cmcm* and *Pnma* in sulfides ALnCuS_3_ (A = Sr, Eu, Ba) clearly, the tolerance factor t′ = [*r*A^2+^] × [*r*Cu^+^] + a × [*r*Ln^3+^]^2^ (a = −0.6, critical value of t′ = 0.28) was applied [10,70]. The experimental data on selenides ALnCuSe_3_ obtained here allow for checking the formula for these compounds. Considering the data on both sulfides and selenides, the adjusted value of the tolerance factor is 0.273. A slight change in the value of t′ confirms the previous assumption that the change in the space group of compounds ALnCuX_3_ is almost independent on the ionic radius of the chalcogen, but depends on the ionic radii of A^2+^, Ln^3+^ and Cu^+^ [70]. We assume that studying new compounds ALnCuX_3_ (X = S, Se; A is an element with an ionic radius in the range of 1.2 Å (*r*Eu^2+^) < (*r*A^2+^) < 1.35 Å (*r*Ba^2+^)), the value of t′ can vary in the range from 0.273 to 0.285.

Thus, our results on the crystal structures of the reported quaternary selenides are in full agreement with the previously established structural types for compounds of the type A^2+^Ln^3+^Cu^+1^X_3_ (A = Eu, Sr, Pb, Ba; X = S, Se) [8]. Furthermore, the formation of the most symmetric structure of the type KZrCuS_3_ with the decreasing of *r*Ln^3+^ and decreasing of the distortion of two-dimensional [LnCuSe_3_] layers in a series of the discussed compounds is also confirmed.

In compounds EuLnCuS_3_, the europium ion is Eu^2+^ [11,68,71]. An insignificant structural difference also anticipates a similarity of charges in EuLnCuX_3_ (X = S, Se). The bond valence sum calculation is frequently used as an efficient tool to estimate the oxidation states of atoms.

The oxidation states of the Eu, Ln and Cu ions in EuLnCuSe_3_ are close to 2, 3 and 1, respectively (Table S7 in the Supplementary Materials). This is in agreement with the data for Eu_2_CuSe_3_ on the presence of both Eu^2+^ and Eu^3+^ [31]. Moreover, the Eu^3+^ ion occupies the same position in Eu^2+^Eu^3+^CuSe_3_ as Ln^3+^ in EuLnCuSe_3_.

### 2.3. Elastic Properties

**Figure 5:**
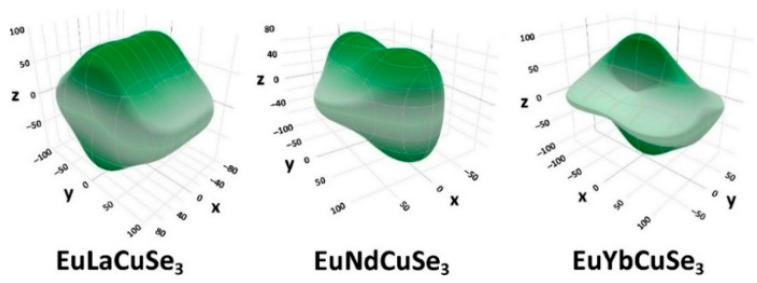
Dependence of the Young’s modulus (GPa) on the direction in the crystals of EuLnCuSe_3_ (Ln = La, Nd, Yb).

Elastic constants of crystals EuLnCuSe_3_ were calculated using the PBE0 functional (Table S8 in the Supplementary Materials), while the bulk modulus, Young’s modulus, and shear modulus were calculated in the Voigt, Reuss and Hill approximations (Table S9 in the Supplementary Materials). The dependence of the Young’s modulus on the direction demonstrates anisotropy of the elastic properties (Figure 5). Calculations predict that the elastic properties of crystals of EuLnCuSe_3_ (Ln = La–Lu) strongly depend on the structural types Ba_2_MnS_3_, Eu_2_CuS_3_ and KZrCuS_3_ (Figure 5, Table S8 in the Supplementary Materials), for example, for the former two structural types C_12_ < C_13_, while C_12_ > C_13_ for the latter structural type (Table S8 in the Supplementary Materials).

The calculated values of the shear modulus and bulk modulus allows the estimation of the Vickers hardness for SrTmCuS_3_ according to empirical formula *H_V_* = 0.92(*G*/*B*)^1.137^ × *G*^0.708^, where *G* is the shear modulus, and *B* is the bulk modulus, obtained from the Hill approximation [72]. It was found that for the structures with space group *Pnma*, the calculated hardness decreases from La to Ho, while for the structures with space group *Cmcm*, the calculated hardness increases from Er to Lu (Table 2). Furthermore, using formula *A^U^* = 5(*G_V_*/*G_R_*) + *B_V_*/*B_R_* − 6 (*G_V_* and *G_R_*, *B_V_* and *R_R_* are the shear and bulk modulus, calculated in the Voigt and Reuss approximations, respectively), we also calculated the so-called “universal” anisotropy index *A^U^* to estimate anisotropy of the elastic properties of EuLnCuSe_3_ [73]. Deviation of the index *A^U^* from zero determines the degree of anisotropic properties of the crystal. It was found that for the crystals of EuLnCuSe_3_ with space group *Pnma*, the value of *A^U^* increases, while for the crystals with space group *Cmcm*, the same value decreases (Table 2).

### 2.4. Phonon, Raman and IR Spectra

**Figure 6:**
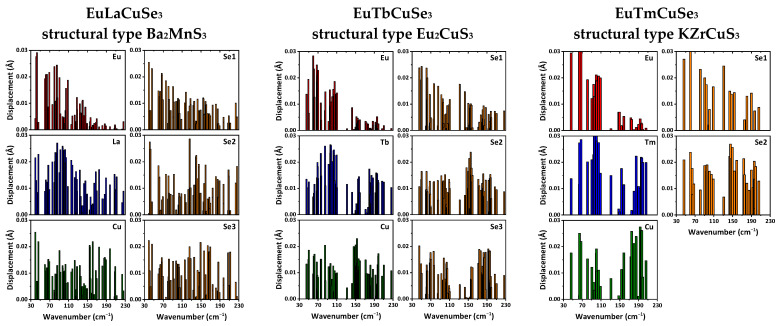
The displacement of ions at phonon modes in EuLnCuSe_3_ (Ln = La, Tb, Tm).

We also applied ab initio calculations with the PBE0 functional to examine wavenumbers and types of the phonon modes. As a result, IR active modes, Raman modes and “silent” modes were revealed (Tables S10–S12 in the Supplementary Materials). Participation of each ion in a particular mode was estimated from the analysis of displacement vectors obtained from these ab initio calculations (Figure 6 and Tables S13–S15 in the Supplementary Materials).

According to the obtained results, the phonon wavenumbers at the Γ-point do not exceed 230 cm^−1^. In this range, not only light ions of selenium and copper but also heavy rare-earth ions are actively involved. In crystals of EuLnCuSe_3_ with a structural type of Ba_2_MnS_3_, the europium ions actively participate in modes with wavenumbers up to about 150 cm^−1^, while for the structural types of Eu_2_CuS_3_ and KZrCuS_3_, the europium ions participate in modes with wavenumbers up to about 110 cm^−1^, respectively (Figure 6). Both the Ln^2+^ and selenium ions are involved in almost all modes for all the reported structural types (Figure 6).

**Figure 7:**
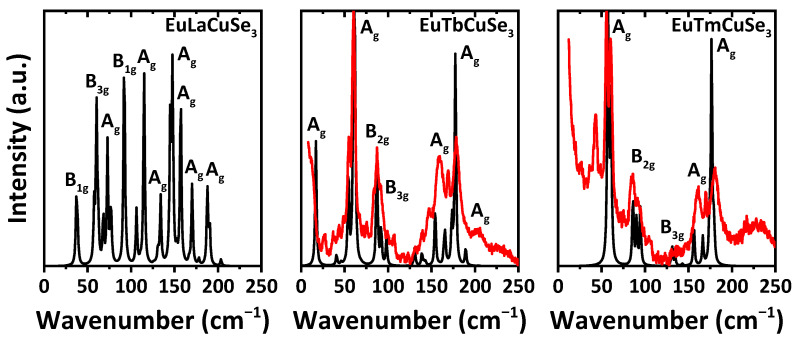
The calculate (black) and experimental (red) Raman spectra for the selected selenides of three different structural types. Calculations were performed at λ_exc_ = 514.5 nm and T = 300 K for EuLaCuSe_3_, and for λ_exc_ = 532 nm and T = 296 K for EuTbCuSe_3_ (*Pnma*) and EuTmCuSe_3_ (*Cmcm*), respectively. The wavenumber scale of the calculated spectra was multiplied by 0.9 to compensate the overestimated results obtained by the applied PBE0 functional.

Unfortunately, contrarily to the corresponding sulfide analogues [9,11], we faced problems collecting Raman spectra for some of the reported selenides, and our numerous attempts failed. This can be explained by the narrower bandgap and sequent high absorption at the wavelengths of excitation and of the scattered Raman signal. As a result, we were able to record the Raman spectra for EuTbCuSe_3_ and EuTmCuSe_3_ (Figure 7). Both the experimental and calculated Raman spectra, obtained using the PBE0 functional, for three typical representatives of each structural types of the reported selenides, namely EuLaCuSe_3_ (structural type Ba_2_MnS_3_), EuTbCuSe_3_ (structural type Eu_2_CuS_3_) and EuTmCuSe_3_ (structural type KZrCuS_3_), exhibit bands exclusively in the region up to about 250 cm^−1^ (Figure 7). Notably, the experimental and calculated spectra of the latter two compounds are pairwise very similar with the most intensive bands at about 60 and 180 cm^−1^, each corresponding to active A_g_ modes. However, the spectrum of EuTbCuSe_3_ contains an additional clearly visible band at about 15 cm^−1^ also due to the A_g_ mode (Figure 7). Both spectra further exhibit bands from B_2g_ and B_3g_ modes. Notably, the experimental spectrum of EuTmCuSe_3_ exhibits an additional clearly defined band at about 45 cm^−1^ (Figure 7). We can tentatively assign the origin of this band to the Raman active mode of the Tm_4_Se_3_O_4_ impurity. Unfortunately, our numerous attempts to calculate the Raman spectrum of Tm_4_Se_3_O_4_ failed and was found to be highly time-consuming, which can be explained by the nonstoichiometric occupancy of the selenium ions.

Interestingly, the calculated spectrum of EuLaCuSe_3_ is characterized by a noticeably larger number of bands (Figure 7). An overwhelming majority of these bands are due to A_g_ modes, with the most intensive one at about 150 cm^−1^, accompanied with two bands at about 60 and 90 cm^−1^, arising from B_3g_ and B_1g_ modes, respectively, and one band at about 115 cm^−1^, corresponding to the A_1g_ mode (Figure 7).

**Figure 8:**
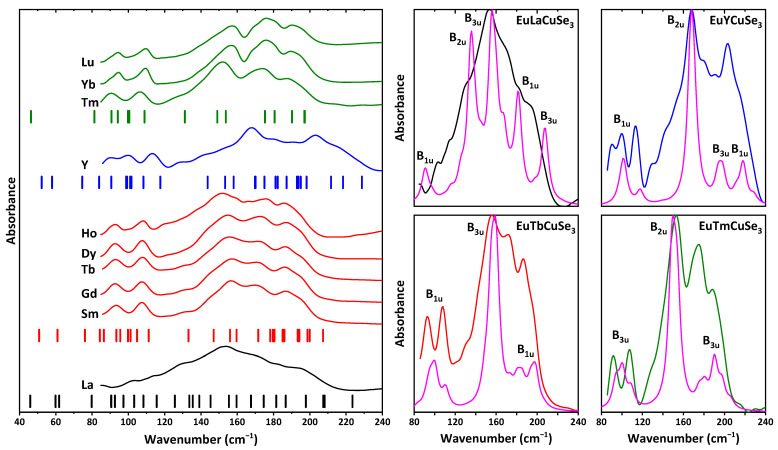
(**left**) IR spectra of EuLnCuSe_3_. Ticks stand for the calculated wavenumbers for EuLaCuSe_3_ (structural type Ba_2_MnS_3_), EuSmCuSe_3_ and EuYCuSe_3_ (structural type Eu_2_CuS_3_) and EuTmCuSe_3_ (structural type KZrCuS_3_) in Table S10 in the Supplementary Materials. (**right**) The calculated (magenta) and experimental IR spectra of EuLaCuSe_3_ (black), EuTbCuSe_3_ (red), EuYCuSe_3_ (blue) and EuTmCuSe_3_ (green).

The structures of EuLnCuSe_3_ were also studied by IR spectroscopy. Notably, all the reported selenides were found to be transparent for IR radiation in the range of 250–4000 cm^−1^. However, a close inspection of the range of 85–250 cm^−1^ revealed a set of absorption bands in the IR spectra of compounds (Figure 8).

Mechanical representation in the center of the Brillouin zone for EuLnCuSe_3_ with the structural types Ba_2_MnS_3_ and Eu_2_CuS_3_ is described as Γ_vibr_ = 12*A_g_* + 6*A_u_* + 6*B*_1*g*_ + 12*B*_1*u*_ + 12*B*_2*g*_ + 6*B*_2*u*_ + 6*B*_3*g*_ + 12*B*_3*u*_. Mechanical representation for the structural type KZrCuS_3_ is the sum of 36 irreducible representations: 5*A_g_* + 2*A_u_* + 4*B*_1*g*_ + 7*B*_1*u*_ + *B*_2*g*_ + 7*B*_2*u*_ + 5*B*_3*g*_ + 5*B*_3*u*_. Notably, the *g* and *u* labeled modes are Raman and IR active, respectively, except for the “silent” *A_u_* modes. Thus, the number of IR active modes is 16 and 27 for the structural types KZrCuS_3_, and Eu_2_CuS_3_ and Ba_2_MnS_3_, respectively.

The experimental IR spectra of the discussed selenides are of three different profiles and in good agreement with the calculated ones (Figure 8). Particularly, the spectra of the structural types KZrCuS_3_ and Eu_2_CuS_3_ (except for EuYCuSe_3_) are very similar, and a set of bands is observed at about 80–210 cm^−1^. This is also supported by the positions of the calculated IR active bands for these selenides, although the total number of these bands differs significantly (Figure 8). The experimental IR spectrum of the yttrium-based selenide exhibits bands in a broader range at about 80–240 cm^−1^. Although the structure of this compound also belongs to the structural type Eu_2_CuS_3_ as for the Sm-, Gd-, Tb-, Dy- and Ho-based derivatives, again the different nature of yttrium (*d*-element) is reflected. EuLaCuSe_3_ is the only described herein selenide of the structural type Ba_2_MnS_3_. Its experimental IR spectrum is, in general, the result of overlapping of closely positioned bands without any large gaps between the IR active vibrations (Figure 8). It should also be noted that all the reported selenides, except for the La-based one, are contaminated by the Ln_4_O_4_Se_3_ [74] or Ln_2_SeO_2_ [75] impurities (see discussion above). Thus, additional bands from the Ln–Se vibrations of the oxyselenides are also expected in the same range as for the quaternary selenides. However, their impact is negligible as evidenced from very weak bands of the oxyselenides Ln–O vibrations found at about 300–600 cm^−1^ [76].

### 2.5. Band Structure and Optical Properites

**Figure 9:**
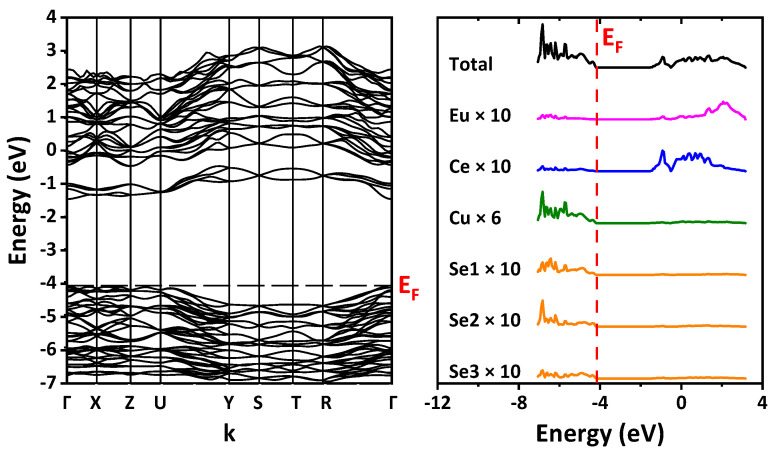

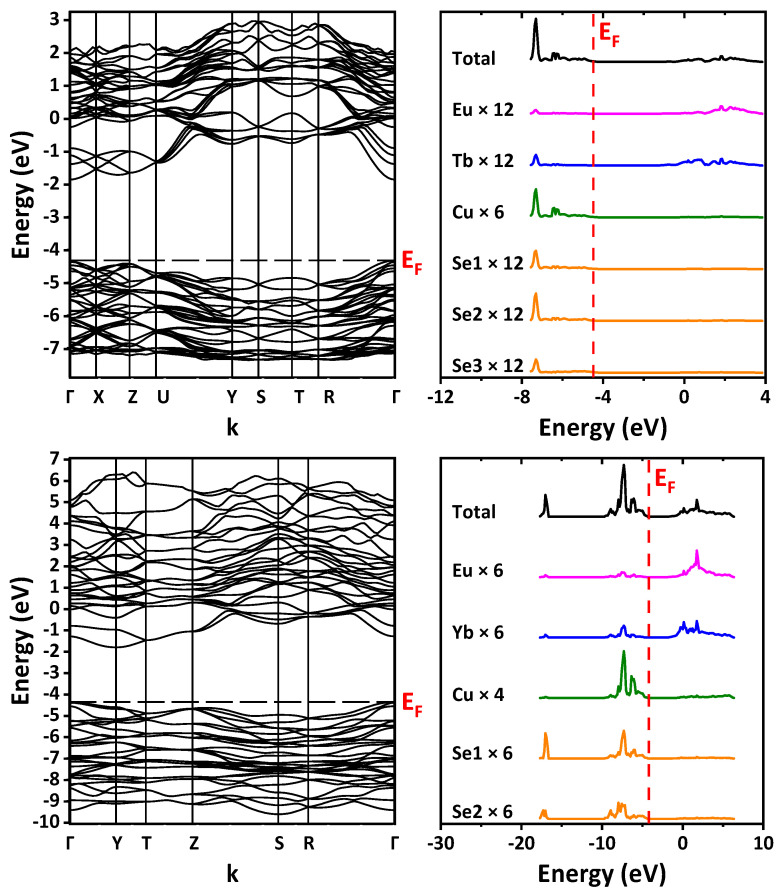
Band structures and the density of states for EuCeCuSe_3_ (**top row**), EuTbCuSe_3_ (**middle row**) and EuYbCuSe_3_ (**bottom row**).

The DFT/B3LYP-calculated band structure and the density of states were examined (Figure 9). The path in the Brillouin zone is plotted through the most highly symmetric points for the orthorhombic lattice. For space group *Pnma* (no. 62), the path is built through Γ–X–Z–U–Y–S–T–R–Γ with the coordinates of points of (0,0,0), (1/2,0,0), (0,0,1/2), (1/2,0,1/2), (0,1/2,0), (1/2,1/2,0), (0,1/2,1/2), (1/2,1/2,1/2), (0,0,0), respectively. For space group *Cmcm* (no. 63), the path is built through Γ–Y–T–Z–S–R–Γ, and the coordinates of points are (0,0,0), (1/2,1/2,0), (1/2,1/2,1/2), (0,0,1/2), (0,1/2,0), (0,1/2,1/2), (0,0,0), respectively. Since for the rare-earth ions pseudopotentials that replaced their core shells, including 4*f*, were used, the band structure does not include 4*f* states. According to calculations, the top of the valence band is formed mainly by the states of copper and selenium, while the bottom of the conduction band is formed by the states of the Ln ion and europium. The band gap was defined as the difference in energy between the top of the valence band and the bottom of the conduction band, namely the HOMO–LUMO value (Table 2 and Figure S3 in the Supplementary Materials). Calculations predict the direct band gap Γ–Γ for EuLnCuSe_3_ with space group *Pnma* (structural types Ba_2_MnS_3_ and Eu_2_CuS_3_) and the indirect band gap Γ–Y for EuLnCuSe_3_ with space group *Cmcm* (structural type KZrCuS_3_). A small jump was also revealed in the band gap value from the structural type Ba_2_MnS_3_ to the structural type Eu_2_CuS_3_ (Figure S3 in the Supplementary Materials).

It is known that, for the same compound, hybrid functionals yield a higher value of the gap width than non-hybrid ones. For example, it was shown that the hybrid functional PBE0 overestimates, while the non-hybrid functional PBE underestimates the gap in some oxides [65]. The same is true for the discussed selenides EuLnCuSe_3_ (Table 2). Furthermore, the non-hybrid PBE functional also yielded an underestimated value of the gap width for selenides as reported earlier (Table 2 and Figure S3 in the Supplementary Materials) [32].

The PBE-based calculation performed in this revealed the gap width close to the results of the PBE calculations reported earlier (Table 2), where the *f*-shell of the rare-earth ions was considered explicitly and was not replaced by a pseudopotential [32]. We also performed a test calculation with the PBE functional for EuLaCuSe_3_, where the *f*-electrons of europium were considered explicitly. The obtained gap of about 1 eV is in good agreement with the reported one [32] and is close to the results, which were obtained by replacing the *f*-shell of europium with a pseudopotential.

The experimental band gaps of EuLnCuSe_3_ were determined from the Kubelka-Munk functions (Figure S4 in the Supplementary Materials). A recent study suggested that use of the simple Kubelka-Munk function may lead to errors in band gap values of the order of several tenths of eV [77]. Landi et al. argue that the Tauc-type modification of the Kubelka-Munk functions, though containing approximations, leads to more correct band gap values. Therefore, we tested both uncorrected (Figure S4 in the Supplementary Materials) and modified Kubelka-Munk functions (Table 2 and Figure S3 in the Supplementary Materials). For some samples, the Kubelka-Munk functions modified for an indirect band gap failed, and the corresponding cells in Table 2 were left empty. Most of the unmodified Kubelka-Munk spectra exhibit a shoulder at longer wavelengths preceding the onset of an evident fundamental absorption. This shoulder is associated with oxyselenide impurities (Table 1) that are known to be semiconductors with the band gap of 1.02–1.46 eV [74]. Thus, to estimate the band gap value of the discussed selenides, an additional correction was applied with respect to impurities [78]. For the Dy- and Sm-based selenides, such a correction was less than 0.01 eV, while no correction was applied for the Y-based derivative since additional data on impurities are required. For the Gd-, Ho-, Tb-, Lu- and Yb-derived compounds, a correction of several hundredths of eV was revealed for the samples containing about 1% of impurities (Table 2).

The obtained values of the band gap are close to those obtained for isostructural sulfides EuLnCuS_3_ (Figure S4 in the Supplementary Materials). The band gap of BaLnCuS_3_ is much higher than that of EuLnCuX_3_ (X = S, Se) [70,79].

Narrowing of the band gap is well-known for the majority of ab initio calculations using the PBE functional. We applied the B3LYP and PBE0 functionals to calculate the electronic band structure, which yielded overestimated band gap values in comparison to the experimental ones, while calculations preformed earlier using the PBE functional and considering *f*-electrons, in general, underestimated the band gap values [32].

The most probable ion that could be responsible for this narrowing is Eu^2+^, which is present in all of the discussed compounds. We suggest that interband transitions between sub-bands originating from partially occupied orbitals of *f*-electrons of Eu^2+^ to, e.g., unoccupied bands originating from *d*-orbitals of the same ion, are not correctly described by a pseudopotential, and these sub-bands are the factor that determines experimental behavior of band gaps (Figure S3 in the Supplementary Materials). More specifically, experimental values of the direct band gap in all selenides containing divalent europium and trivalent lanthanide with *f*-electrons vary from 1.87 eV to 1.97 eV for the *Pnma* space group, and from 2.02 eV to 2.1 eV for the *Cmcm* space group (Tm, Lu).

The B3LYP-based calculation with the europium pseudopotential predicts direct band gaps in the range 2.38–2.63 eV and 2.52–2.59 eV for *Pnma* and *Cmcm* space groups, respectively. An indirect band gap for the *Cmcm* space group is predicted to be narrower than the direct one in contrast to the *Pnma* space group. However, for consistency, we compare direct band gaps produced by stronger direct transitions for all crystals. The discrepancy between the B3LYP-based calculations and experimental values of the order of 0.6 eV is ascribed to a lower position of excited *d*-bands of europium in comparison with the B3LYP calculation. This explanation is valid for all the discussed compounds except for the La-, Y- and Yb-derived ones, which exhibit much narrower experimental band gaps. For the Yb containing selenide, the narrower band gap can be explained as similar to BaYbCuS_3_ [79]. The Yb^3+^ ion with the *f*^13^-electronic configuration is characterized by a low-lying charge transfer transition to the *f*^14^-configuration with enhanced stability. In selenides, this charge transfer transition is expected to lie at even lower energies than in sulfides, resulting in additional narrowing of the EuYbCuSe_3_ band gap with respect to calculations. To explain the narrowing of the band gap of compounds where Ln^3+^ completely lack *f*-electrons, i.e., La and Y are more challenging, one may expect that the lack of *f*-electrons results in certain structural changes that lead to enhancement of the crystal field acting upon europium ions and sequent narrowing of the band gap. Let us compare, e.g., a local environment of the europium ion in EuYCuSe_3_ and in EuHoCuSe_3_ with the ionic radius of Ln^3+^ closest to that of Y^3+^. The europium ion is coordinated by seven selenium ions at distances of the order of 3 Å. Specifically, one of the selenium ions is positioned at a much longer distance of about 3.4–3.5 Å for the Ho- and Y-based selenides, respectively. Thus, asymmetry of the crystal field for EuYCuSe_3_ is the largest one. Hypothetically, it can be the reason for the narrowing of the EuYCuSe_3_ band gap. However, since the increase of asymmetry is rather small, this explanation must be considered as a hypothesis.

### 2.6. Magnetic Properties

**Figure 10:**
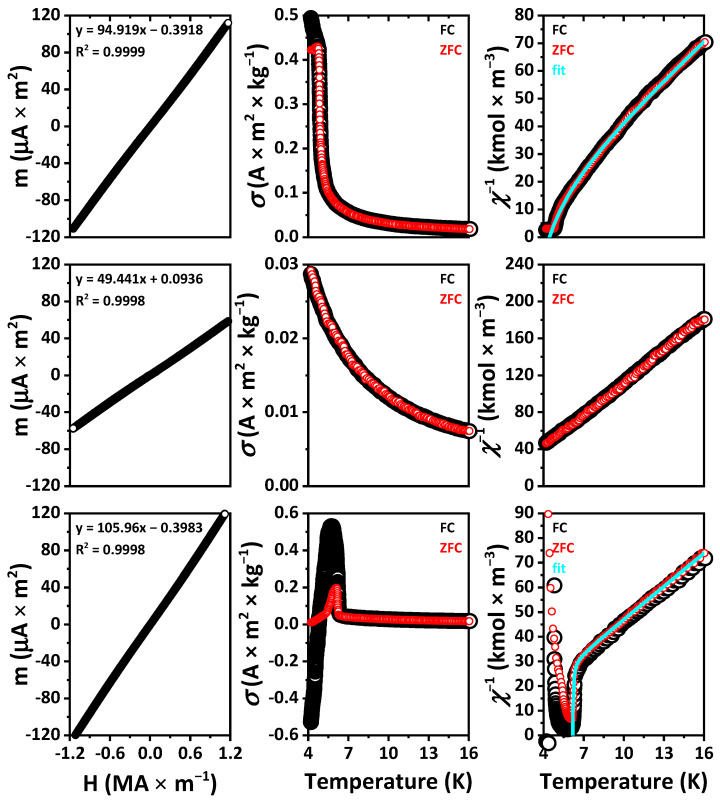
Field-dependent magnetic moments at 296 K (**left**), and temperature-dependent specific magnetization (middle) and reciprocal magnetic susceptibility (**right**) of EuTmCuSe_3_ (**top row**), EuLaCuSe_3_ (**middle row**) and EuHoCuSe_3_ (**bottom row**), respectively, at 10 Oe. The measurements of low-temperature magnetization were performed in the zero-field cooled (ZFC) and nonzero-field cooled (FC) modes. Fit line shows approximation by the Néel model of a ferrimagnet [80].

The external field dependent magnetic moments of the samples at room temperature exhibit a linear plot, corresponding to the Curie law for paramagnets and differ only in the slope of the line (Figure 10 and Figure S5 in the Supplementary Materials).

The calculated values of μ and *C* for EuYCuSe_3_, EuLaCuSe_3_ and EuLuCuSe_3_ are the same since the cations Y^3+^, La^3+^ and Lu^3+^ are diamagnetic, and the final magnetic properties of these selenides are exclusively associated with the Eu^2+^ cation. The experimental values of the effective magnetic moments of EuYCuSe_3_ and EuLaCuSe_3_ are equal within the error and differ from the calculated ones by about 1%, while the experimental magnetic moment of EuLuCuSe_3_ is about 5.5% larger in comparison to the calculated one (Table 3). For the other selenides, the relative deviations of the calculated values of the magnetic moments from the calculated ones do not exceed 3%. As for the corresponding Curie constants, this value of deviation is as twice as large due to the quadratic dependence on the values of the effective magnetic moments.

Plots of both specific magnetization and reciprocal magnetic susceptibility of EuLaCuSe_3_ and EuLuCuSe_3_ at 4.2–20 K indicate that, upon cooling down to 4.2 K, the compounds remain paramagnetic with the corresponding (asymptotic) Curie temperature θ_p_ = 0.2 and 3.1 K, respectively (Figure 10 and Figure S5 in the Supplementary Materials). At these temperatures, the samples are most likely to be ferromagnetic. This assumption is supported by the presence of one type of magnetic ions in the compounds (Eu^2+^), the coincidence of the data for the FC and ZFC measurements and the linearity of the temperature dependent reciprocal susceptibility down to the lowest temperatures.

The magnetic behavior of EuYCuSe_3_ is similar to that of EuLaCuSe_3_ and EuLuCuSe_3_. However, below 12 K, the FC and ZFC curves slightly diverge, although there are no noticeable points in these dependences (Figure S5 in the Supplementary Materials). The paramagnetic Curie temperatures were found to be 4.2 and 3.3 K for FC and ZFC, respectively. The second value is more reliable since the dependence in this case is more linear. Probably, a transition to a ferromagnetic state occurs at this point. The thermomagnetic dependences for EuYbCuSe_3_ are similar with the FC and ZFC curves being less diverged, and the transition to a ferromagnetic state occurs, most likely, very close to 4 K.

Curves for EuTbCuSe_3_, EuDyCuSe_3_ (ZFC), EuHoCuSe_3_ and, to a lesser extent, EuTmCuSe_3_ with decreasing temperatures exhibit an abrupt drop in the linear dependence of the reciprocal susceptibility, characteristic of ferrimagnets (Figure 10 and Figure S5 in the Supplementary Materials). Paramagnetic Curie temperatures, calculated from linear sections of the curves, are negative except for EuTmCuSe_3_. This testifies to the antiparallel coordination of the magnetic sublattices. The approximation of these curves at temperatures above the points of local minima *χ*^−1^, according to the Néel theory for ferrimagnets, reveals the data, including the Néel temperatures, gathered in Table 4.

The decreasing of the *χ*^−1^ values of EuDyCuSe_3_ and EuTbCuSe_3_ in the FC mode in comparison to the ZFC mode at low temperatures corresponds to an increase in the magnetic moment of the sample cooled in the presence of an external field. Apparently, when heated above the Néel point, the effect of this initial magnetization persists up to some higher temperatures. A similar explanation can be proposed for the divergence of the FC and ZFC curves of EuYCuSe_3_ and EuYbCuSe_3_, discussed above, although the temperature did not decrease below the points of magnetic transitions. The positive value of the paramagnetic Curie point calculated for EuTmCuSe_3_ contradicts the conclusion about ferrimagnetic ordering, but this value is rather close to zero.

The magnetization of EuHoCuSe_3_ below the Néel point looks significantly different, and its value is negative at temperatures from 4.2 to 4.8 K. This recently attracted increased interest for possible practical applications [81,82]. Several mechanisms of this phenomenon are considered: negative exchange coupling among ferromagnetic sublattices, negative exchange coupling among canted antiferromagnetic sublattices, negative exchange coupling among ferromagnetic/canted antiferromagnetic and paramagnetic sublattices, imbalance of spin and orbital moments, interfacial exchange coupling between ferromagnetic and antiferromagnetic phases [81]. Similar temperature dependences of the reciprocal susceptibility were obtained for (Tm_0.8_Mn_0.2_)MnO_3_ [82]. It was established that the negative magnetization is determined by the first mechanism. Most likely, EuHoCuSe_3_ exhibits similar properties and belongs to the N-type ferrimagnets according to Néel [80].

The magnetic properties of EuYCuSe_3_ and EuLnCuSe_3_ (Ln = La, Tb, Dy, Ho, Tm, Yb, Lu) correlate well with the properties of isostructural sulfides (Table 5) [9,10,68].

## 3. Materials and Methods

### 3.1. Materials

Ln_2_O_3_ (Ln = La, Sm, Eu, Gd, Dy, Ho, Y, Tm, Yb, Lu; 99.9%) and Tb_4_O_7_ (99.9%) were purchased from the Uralredmet manufacture (Verkhnyaya Pyshma, Russia). Elementary selenium (extra-pure grade, 17-4) and CuSO_4_·5H_2_O (pure for analysis) were purchased from the Lenreactiv, CJSC (Saint Petersburg, Russia). Elementary copper (99.9%) was obtained from SZB Tsvetmet, OJSC (Saint Petersburg, Russia). Argon (99.998%) was purchased from Kislorod-servis (Yekaterinburg, Russia), concentrated nitric acid (extra-pure grade, 18-4 all-Union State Standard 11125-84) was purchased from Chemreaktivsnab, CJSC (Ufa, Russia). Activated charcoal was obtained from «Tyumenskie sistemy vodoochistki» Ltd. (Tyumen, Russia).

### 3.2. Synthesis

Powdered samples of EuLnCuSe_3_ were prepared by reductive selenidation of the oxide mixtures produced by thermolysis of the co-crystallized metal nitrates. Metallic copper fragments were mechanically cleaned, treated with alcohol and dried at room temperature. Oxides of rare earth elements were annealed in a quartz glass in a muffle furnace at 1070 K for 5 h, while Tb_4_O_7_ was annealed at 470 K to keep its chemical composition and avoid stepwise transformation.

The parent reagents were calculated based on the stoichiometric ratio of metals in EuLnCuSe_3_ (1:1:1). Starting materials were dissolved in 65% aqueous HNO_3_ (100 mL) under vigorous stirring upon heating to 330–350 K. The resulting solution was slowly evaporated, followed by the co-crystallization of nitrates. The dry residue was transferred into a quartz glass and subjected to thermolysis at 1070 K for 5 h. The baked mass was ground to a size of about 30–50 µm.

Reductive selenidation was carried out in a flow of H_2_ (pressure 0.004 MPa) and H_2_Se, obtained by the interaction of hydrogen with elemental selenium at 770 K. At the outlet of the reaction zone, an excess of H_2_Se was passed through 200–300 g of activated charcoal. The resulting gas was bubbled through a 0.1 M CuSO_4_ solution. Activated charcoal was replaced by a fresh portion once flakes of Cu_2_Se (*K*_SP_ = 60.6 [83]) appeared. Selenidation was carried out with periodic grinding of the resulting product at 970–1040 K for 2.5–12 h and at 1170 K for 6–12 h.

The resulting products were examined by SEM-EDX, and the obtained data are collected in Table 6.

### 3.3. Methods

The powder X-ray diffraction data were collected at room temperature with a ДPOH 7 (Burevestnik, Saint Petersburg, Russia) powder diffractometer (Cu-K_α_ radiation, graphite monochromator). The step size of 2θ was 0.02°, and the counting time was 13–50 s per step. The crystal structures of EuLnCuSe_3_ (Ln = La, Sm, Gd, Tb, Dy, Ho, Y, Tm, Yb, Lu) were refined by the derivative difference minimization (DDM) method [84] in the anisotropic approximation for all atoms. The data for isostructural sulfides Ba_2_MnS_3_ [85], Eu_2_CuS_3_ [71] and KZrCuS_3_ [23] were used as initial structural models. The effects of preferred orientation, anisotropic broadening of peak and sample surface roughness and displacement were taken into account during refinement. Crystal structures were visualized in the program package Diamond 3 [86]. The bond valence method was applied to estimate the oxidation states of atoms [87]. CCDC 2125819–2125828 contain the supplementary crystallographic data. These data can be obtained free of charge via https://www.ccdc.cam.ac.uk/structures (accessed on 23 December 2021) or from the Cambridge Crystallographic Data Centre, 12 Union Road, Cambridge CB2 1EZ, UK; fax: (+44)-1223-336-033; or e-mail: deposit@ccdc.cam.ac.uk.

Scanning electron microscopy (SEM) was performed on a JEOLJSM-6510 LV (JEOL Ltd., Tokyo, Japan) equipped with an energy dispersive spectrometer.

The low-temperature (4.2–20 K) magnetic susceptibilities of EuLnCuSe_3_ (Ln = La, 0.050 g; Tb, 0.070 g; Dy, 0.090 g; Ho, 0.075 g; Y, 0.060 g; Tm, 0.155 g; Yb, 0.085 g and Lu, 0.055 g) were studied on a SQUID magnetometer (Kirensky Institute of Physics, Krasnoyarsk, Russia) [88,89] in a 10 Oe (796 A m^−1^) magnetic field. The measurements of low-temperature magnetization were performed in the zero-field cooled (ZFC) and nonzero-field cooled (FC) modes. The powdered samples of EuLnCuS_3_ (Ln = La, 0.0897 g; Sm, 0.0522 g; Gd, 0.0637 g; Tb, 0.0637 g; Dy, 0.0464 g; Ho, 0.0738 g; Y, 0.0637 g; Tm, 0.0918 g; Yb, 0.0601 g and Lu, 0.0600 g) were tightly packed into the polyvinylchloride container of 4.2 mm in diameter and 5.6 mm height with the lid. The room-temperature magnetic properties of EuLnCuSe_3_ were studied on a vibrating sample magnetometer with a Puzey electromagnet [90]. The magnetic field was varied in the range −15 ÷ 15 kOe (−1.2 ÷ 1.2 MA m^−1^) with a step of 20–100 Oe (1.59–7.96 kA m^−1^). The magnetometer signal from the container and the lid was measured separately and then subtracted from the total signal.

The Fourier-transform infrared (FTIR) absorption spectra in the range of 85–675 cm^−1^ were recorded on a VERTEX 80v FT-IR spectrometer (Bruker OJSC, Germany). The samples were ground in an agate mortar and then mixed with ultrahigh molecular weight polyethylene (Mitsui Petrochemical Ltd., Tokyo, Japan) and pressed into pellets of about 0.26 mm thickness and of 13 mm diameter. A Globar was used for the light source, and it was equipped with a Bruker multilayer beamsplitter and RT-DTGS FIR as a detector. The attenuated total reflectance infrared (ATR-IR) absorption spectra in the range of 400–4000 cm^−1^ were recorded on a Cary 630 FTIR spectrometer (Agilent Technologies Inc., Santa Clara, CA, USA) equipped with an ATR attachment and a DTGS detector.

The diffuse reflectance spectra were recorded on a UV-2600 spectrophotometer (Shimadzu OJSC, Tokyo, Japan) equipped with an ISR-2600Plus attachment with the photomultiplier PMT of the R-928 type and InGaAs detectors. BaSO_4_ (99.8%) was used as a standard.

Gaseous hydrogen was obtained on a SPEKTR 16 4D hydrogen generator (Spektr Ltd., Petrozavodsk, Russia). The temperature in the electric heating furnaces used for synthesis was controlled using a Termodat-16K6 (InSAT Ltd., Moscow, Russia) temperature controller with a chromel-alumel thermocouple.

The experimental Raman spectra of EuLnCuSe_3_ compounds were collected in backscattering geometry, using a triple monochromator Horiba JobinYvon T64000 Raman spectrometer (Horiba Ltd., Tokyo, Japan) operating in subtractive mode. The spectral resolution for the recorded Stokes-side Raman spectra was better than 2.5 cm^−1^ (this resolution was achieved by using gratings with 1800 grooves mm^−1^ and 100 μm slits). The single-mode radiation at 532 nm from the Spectra-Physics Excelsior laser was used as an excitation light source, the power on the sample being 1 mW.

### 3.4. DFT Calculations

The density functional theory (DFT) calculations were carried out using the PBE0 and B3LYP hybrid functionals, which consider both local and nonlocal Hartree-Fock exchanges. The calculations were performed using the CRYSTAL17 program designed to simulate periodic structures [91]. For Eu^3+^, the ECP53MWB quasi-relativistic pseudopotential was used with the attached valence basis set ECP53MWB [92]. For Ln^3+^, the ECP*n*MWB quasi-relativistic pseudopotential was used with the attached valence basis set ECP*n*MWB-I (*n* = *Z* − 1; *Z* is an atomic number) [92]. Thus, the inner shells of the rare-earth ion, including 4*f*, were replaced by a pseudopotential. To describe the outer shells 5*s*^2^5*p*^6^, involved in chemical bonding, valence basis sets were used. Such an approach makes it possible to reconstitute both the lattice structure and dynamics in the compounds that have a lanthanide ion sublattice successfully [10]. For Y^3+^, the ECP*28*MHB pseudopotential was used with the attached valence basis set ECP*28*MWB [92]. For copper and selen, we used full-electron basis sets, known in the CRYSTAL program site as Cu_86-4111(41D)G_doll_2000 and Se_976-311d51G_towler_1995, respectively [91]. Gaussian primitives with orbital exponent values less than 0.1 were removed from basis sets since these calculations are periodic. The exponent in the outer *p*-orbital of the selen basis set was set to 0.1742. The exponent in the outer diffuse *s*-, *p*- and *d-* orbitals of the yttrium valence basis set was set to 0.1312. The accuracy of calculation of the self-consistent field and the two-electron integrals was set at 10^−9^ a.u. and 10^−8^, respectively. Integration over the Brillouin zone was carried out according to the Monkhorst-Pack scheme with a grid of *k*-points equal to 8 × 8 × 8.

The sequence of the DFT calculations was as follows: the crystal structure was first optimized followed by the calculation of the phonon spectrum at the Γ point or the elastic constants.

## 4. Conclusions

In summary, we successfully fabricated novel quaternary selenides of the Eu^+2^Ln^+3^Cu^+1^Se_3_ (Ln = La, Sm, Gd, Tb, Dy, Ho, Y, Tm, Yb and Lu) composition through reductive selenidation of the oxide mixtures, produced by thermolysis of the co-crystallized metal nitrates. The applied synthetic procedure allowed a significant decrease in the reaction time and temperature, and an increase in the yield of the final product in comparison to the recently reported procedure [60]. Crystal structures of the resulting compounds were elucidated by powder X-ray diffraction. As a result, the structures were found to belong to orthorhombic space groups *Pnma* (structural type Ba_2_MnS for EuLaCuSe_3_ and structural type Eu_2_CuS_3_ for EuLnCuSe_3_, where Ln = Sm, Gd, Tb, Dy, Ho and Y) and *Cmcm* (structural type KZrCuS_3_ for EuLnCuSe_3_, where Ln = Tm, Yb and Lu). These space groups were delimited based on the tolerance factor t′, while the formation of three structural types was additionally probed by vibrational spectroscopy and further supported by ab initio calculations with hybrid functionals. It was also established that the revealed structural types for a series of the obtained selenides are the same as for the previously reported sulfide analogues.

According to calculations, the elastic properties change abruptly from one structural type to another. The wavenumbers and types of fundamental vibrations were determined from ab initio calculations, and the experimental IR spectra were interpreted.

Optical properties of the reported compounds EuLnCuSe_3_ were studied by diffuse reflectance spectroscopy. The corresponding band gaps are determined by the interconfigurational transitions between sub-bands originating from the occupied *f*-orbitals and vacant *d*-orbitals of the Eu^2+^ ion.

Field-dependent magnetic properties of the discussed selenides were also revealed by SQUID measurements. The experimental Curie constants and effective magnetic moments at room temperature are very close to calculated ones. Phase transitions to the ferrimagnetic state were established for EuTbCuSe_3_, EuDyCuSe_3_, EuHoCuSe_3_ and EuTmCuSe_3_ with the transition temperatures varying from 4.5 to 6.2 K. For EuHoCuSe_3_, the effect of negative magnetization, which is characteristic of an N-type ferrimagnet according to the Néel’s classification, was found at temperatures below 4.8 K. A comparison of the magnetic properties of quaternary selenides EuLnCuSe_3_ with isostructural sulfides showed a high degree of similarity.

Finally, we believe that a series of the reported selenides together with the revealed properties will be of value for the fabrication of novel materials with properties of interest.

## Figures and Tables

**Table 1:** Experimental details for the structures of EuLnCuSe_3_ (Ln = La, Sm, Gd, Tb, Dy, Ho, Y, Tm, Yb, Lu).

	**EuLaCuSe_3_**	**EuSmCuSe_3_**	**EuGdCuSe_3_**	**EuTbCuSe_3_**	**EuDyCuSe_3_**
Space group	*Pnma*	*Pnma*	*Pnma*	*Pnma*	*Pnma*
Structural type	Ba_2_MnS_3_	Eu_2_CuS_3_	Eu_2_CuS_3_	Eu_2_CuS_3_	Eu_2_CuS_3_
*a* (Å)	8.4659(5)	10.7634(5)	10.6746(11)	10.6027(15)	10.5628(8)
*b* (Å)	4.2140(3)	4.1138(2)	4.0966(4)	4.0843(6)	4.0768(3)
*c* (Å)	16.6636(11)	13.3860(7)	13.3993(14)	13.3926(19)	13.3915(11)
*V* (Å^3^)	594.47(7)	592.71(5)	585.94(11)	579.97(14)	576.67(8)
*D_calc_* (g cm^−3^)	6.60401	6.75257	6.90762	6.99871	7.07976
*R* factors	*R*_DDM_ = 0.058	*R*_DDM_ = 0.040	*R*_DDM_ = 0.060	*R*_DDM_ = 0.042	*R*_DDM_ = 0.058
	*R*_exp_ = 0.053	*R*_exp_ = 0.036	*R*_exp_ = 0.042	*R*_exp_ = 0.039	*R*_exp_ = 0.053
	*R*_Bragg_ = 0.044	*R*_Bragg_ = 0.026	*R*_Bragg_ = 0.031	*R*_Bragg_ = 0.028	*R*_Bragg_ = 0.024
Impurity	–	1% SmCuSeO	1.2% Gd_4_Se_3_O_4_	1.3% Tb_2_SeO_2_	2.8% Dy_4_Se_3_O_4_
	**EuHoCuSe_3_**	**EuYCuSe_3_**	**EuTmCuSe_3_**	**EuYbCuSe_3_**	**EuLuCuSe_3_**
Space group	*Pnma*	*Pnma*	*Cmcm*	*Cmcm*	*Cmcm*
Structural type	Eu_2_CuS_3_	Eu_2_CuS_3_	KZrCuS_3_	KZrCuS_3_	KZrCuS_3_
*a* (Å)	10.5182(8)	10.5659(13)	4.05721(17)	4.0490(2)	4.0434(2)
*b* (Å)	4.0714(3)	4.0800(5)	13.3784(6)	13.3699(7)	13.3694(6)
*c* (Å)	13.3898(10)	13.4006(16)	10.4477(5)	10.4124(6)	10.3954(5)
*V* (Å^3^)	573.40(8)	577.69(12)	567.09(4)	563.67(5)	561.94(5)
*D_calc_* (g cm^−3^)	7.14826	6.22125	7.27467	7.36674	7.41257
*R* factors	*R*_DDM_ = 0.057	*R*_DDM_ = 0.061	*R*_DDM_ = 0.046	*R*_DDM_ = 0.047	*R*_DDM_ = 0.045
	*R*_exp_ = 0.038	*R*_exp_ = 0.050	*R*_exp_ = 0.038	*R*_exp_ = 0.040	*R*_exp_ = 0.040
	*R*_Bragg_ = 0.029	*R*_Bragg_ = 0.041	*R*_Bragg_ = 0.021	*R*_Bragg_ = 0.022	*R*_Bragg_ = 0.019
Impurity	2.2% Ho_2_SeO_2_	2.2% Y_2_SeO_2_	4.9% Tm_4_Se_3_O_4_	4.1% Yb_4_Se_3_O_4_	4.3% Lu_4_Se_3_O_4_

**Table 2:** Bulk modulus (*B*), shear modulus (*G*), Vickers hardness (*H_V_*), universal anisotropy index (*A^U^*), calculated (Calc.) and experimental (Ex.) values of the band gap (BG) for EuLnCuSe_3_ (Ln = La, Ce, Pr, Nd, Sm, Gd, Tb, Dy, Ho, Y, Er, Tm, Yb, Lu).

	La	Ce	Pr	Nd	Sm	Gd	Tb	Dy	Ho	Y	Er	Tm	Yb	Lu
Space group	*Pnma*										*Cmcm*			
Structural type	Ba_2_MnS_3_		Eu_2_CuS_3_								KZrCuS_3_			
*B* (GPa)	77.2	77.6	66.6	67.1	68.2	68.5	69.1	69.1	69.2	70.0	70.9	71.4	71.7	72.0
*G* (GPa)	35.6	31.6	32.0	31.9	31.2	30.1	30.3	27.8	24.3	30.4	25.1	26.9	28.1	28.8
*H_V_* (GPa)	4.8	4.9	4.7	4.6	4.3	4.0	4.0	3.4	2.7	4.0	2.8	3.1	3.4	3.5
*A^U^*	0.29	0.31	0.59	0.67	1.07	1.62	1.68	3.04	6.39	1.92	6.46	4.69	3.83	3.45
Calc. BG_B3LYP_ (eV) ^1^	2.63	2.63	2.38	2.38	2.42	2.45	2.48	2.49	2.51		2.52	2.54	2.56	2.59
Calc. BG_PBE0_ (eV) ^1^	2.78	2.79	2.50	2.51	2.53	2.56	2.58	2.59	2.61		2.62	2.65	2.67	2.69
Calc. BG_PBE_ (eV) ^1^	1.25	1.25	1.00	1.01	1.04	1.06	1.07	1.08	1.10	1.04	1.12	1.13	1.14	1.15
Calc. BG_PBE_ (eV) [32]	0.94	0.91	0.95	0.98	1.04	0.96	1.11	1.02	1.04	1.07	1.06	1.09	–	1.13
Exp. BG_indirect_ (eV) ^1^	1.19	–	–	–	–	1.01	0.93	0.93	–	0.80	–	1.24, 1.70	–	2.06
Exp. BG_Kubelka-Munk_ (eV) ^1^	1.35	–	–	–	1.04, 1.76	1.11, 1.82	0.93, 1.88	1.09, 1.60	1.14, 1.87	1.03	–	1.16	–	1.38
Exp. BG_direct_ (eV) ^1^	1.54	–	–	–	1.95	2.01	1.97	1.87	2.05	1.19	–	1.07	–	2.09

^1^ This work.

**Table 3:** Magnetic characteristics for EuLnCuSe_3_ (Ln = La, Sm, Gd, Tb, Dy, Ho, Y, Er, Tm, Yb, Lu).

	La	Sm	Gd	Tb	Dy	Ho	Y	Tm	Yb	Lu
Space group	*Pnma*							*Cmcm*		
Structural type	Ba_2_MnS_3_	Eu_2_CuS_3_						KZrCuS_3_		
*χ*·10^6^ (m^3^ mol^−1^)	0.326	0.330	0.628	0.887	0.884	0.886	0.325	0.644	0.430	0.373
Exp. *μ*_296K_ (μ_B_)	7.86	7.87	10.86	12.90	12.94	12.95	7.85	10.99	9.00	8.38
Exp. *μ*_15K_ (μ_B_)	7.5	–	–	10.7	11.0	12.3	10.9	12.0	8.8	14.0
Calc. *μ* (μ_B_)	7.937	7.982	11.225	12.550	13.279	13.248	7.937	10.962	9.142	7.937
Exp. *C*_296K_ (K m^3^ kmol^−1^)	0.0970	0.0972	0.1853	0.2616	0.2631	0.2634	0.0969	0.1899	0.1272	0.1102
Exp. *C*_15K_ (K m^3^ kmol^−1^)	0.09	–	–	0.19	0.19	0.24	0.19	0.22	0.12	0.32
Calc. *C* (K m^3^ kmol^−1^)	0.09900	0.1001	0.1980	0.24750	0.27709	0.27578	0.09900	0.18883	0.13133	0.09900
Arrangement ^1^	Ferro	–	–	Ferri	Ferri	Ferri	Ferro	Ferri	Ferro	Ferro
*θ*_p_ (K)	0.2	–	–	−1.3	−0.7	−0.4	3.3	1.1	4.5	3.1

^1^ Ferro = ferromagnetic, Ferri = ferrimagnetic.

**Table 4:** Calculation model parameters for ferrimagnetic EuLnCuSe_3_ (Ln = Tb, Dy, Ho, Tm) ^1^.

	Tb	Dy	Ho	Tm
Space group	*Pnma*			*Cmcm*
Structural type	Eu_2_CuS_3_			KZrCuS_3_
*C* (K m^3^ kmol^−1^)	0.19	0.19	0.24	0.22
1/*χ*_0_ (kmol m^−3^)	13	12	2.8	3.8
*σ* (K m^3^ kmol)	7.5	29	1.0	55
*θ* (K)	5.8	4.7	6.1	2.1
*T_c_* (K)	6.0	5.5	6.2	4.5

^1^ Formula applied for the model: $\frac{1}{\chi }=\frac{T}{C}+\frac{1}{\chi_{0}}\;–\;\frac{\sigma }{T\;–\;\theta }$; $T_{c}=\frac{1}{2}\left(\theta \;–\;\frac{C}{\chi_{0}}+\sqrt{{\left(\theta \;–\;\frac{C}{\chi_{0}}\right)}^{2}+4C\left(\frac{\theta }{\chi_{0}}+\sigma \right)}\right)$.

**Table 5:** Comparison of the magnetic characteristics for EuLnCuX_3_ (Ln = La, Tb, Dy, Ho, Y, Er, Tm, Yb, Lu; X = S, Se).

	La	Tb	Dy	Ho	Y	Er	Tm	Yb	Lu
Space group	*Pnma*					*Cmcm*			
Structural type	Ba_2_MnS_3_	Eu_2_CuS_3_				KZrCuS_3_			
*T_c_* for X = S (K)	2.4 [10]	4.9 [68]	4.6 [68]	4.8 [10]	4.5 [68]	4.8 [11]	4.8 [68]	5.5 [68]	5.4 [68]
*T_c_* for X = Se (K)	~1	6.0	5.5	6.2	~3	4.7 [60]	4.5	~4.3	~3
Arrangement for X = S ^1^	Ferro	Ferri	Ferri	Ferri	Ferro	Ferri	Ferri	Ferro	Ferro
Arrangement for X = Se ^1^	Ferro	Ferri	Ferri	Ferri	Ferro	Ferri	Ferri	Ferro	Ferro

^1^ Ferro = ferromagnetic, Ferri = ferrimagnetic.

**Table 6:** The calculated and found elemental analysis data for the reported selenides obtained using SEM-EDX.

Compound (Mass)	Calculated (%)					Found (%)				
	Eu	Ln	Cu	Se	O	Eu	Ln	Cu	Se	O
EuLaCuSe_3_ (591.29)	25.70	23.49	10.75	40.06	–	25.65	23.40	10.72	40.23	–
EuSmCuSe_3_ (602.75)	25.21	24.95	10.54	39.30	–	25.44	24.68	10.64	39.19	0.05
EuGdCuSe_3_ (609.64)	24.93	25.79	10.42	38.86	–	24.65	26.31	10.29	38.67	0.08
EuTbCuSe_3_ (611.31)	24.86	26.00	10.39	38.75	–	24.55	26.65	10.25	38.45	0.10
EuDyCuSe_3_ (614.89)	24.71	26.43	10.34	38.52	–	23.99	27.62	10.05	38.15	0.19
EuHoCuSe_3_ (617.32)	24.62	26.72	10.29	38.37	–	24.05	27.76	10.07	37.96	0.16
EuYCuSe_3_ (541.29)	28.07	16.42	11.74	43.76	–	27.47	17.43	11.47	43.38	0.25
EuTmCuSe_3_ (621.32)	24.46	27.19	10.23	38.12	–	23.24	29.24	9.72	37.48	0.32
EuYbCuSe_3_ (625.43)	24.30	27.67	10.16	37.87	–	23.31	29.37	9.75	37.31	0.26
EuLuCuSe_3_ (627.35)	24.22	27.89	10.13	37.76	–	23.16	29.68	9.70	37.18	0.28

## Data Availability

All the data supporting the conclusions is included within the manuscript and is available on request from the corresponding authors.

## Supplementary Materials

The following supporting information can be downloaded at: https://www.mdpi.com/article/10.3390/ijms23031503/s1.
