# Supplementary material for: Quaternary Selenides EuLnCuSe3: Synthesis, Structures, Properties and In Silico Studies
Source: Int J Mol Sci. 2022 Jan 28;23(3):1503. doi: 10.3390/ijms23031503 (PMC8836063; doi:10.3390/ijms23031503)
Supplement: Supplementary file 1 [file ijms-23-01503-s001.zip › ijms-1548505-supplementary.pdf]

Supplementary Materials

Quaternary Selenides $EuLnCuSe_3$: Synthesis, Structures, Properties and In Silico Studies

Maxim V. Grigoriev [1], Leonid A. Solovyov [2], Anna V. Ruseikina [3,*], Aleksandr S. Aleksandrovsky [4,5], Vladimir A. Chernyshev [6], Dmitriy A. Velikanov [5], Alexander A. Garmonov [7], Maxim S. Molokeev [5,8,9], Aleksandr S. Oreshonkov [5,10], Nikolay P. Shestakov [5], Alexey V. Matigorov [11], Svetlana S. Volkova [3], Evgeniy A. Ostapchuk [1], Alexander V. Kertman [3], Thomas Schleid [12] and Damir A. Safin [3,13,14,*]

[1] Laboratory of Theory and Optimization of Chemical and Technological Processes, University of Tyumen, 625003 Tyumen, Russia; maxgrigmvv@yandex.ru (M.V.G.); ostapchuk_evgeniy@list.ru (E.A.O.)

[2] Institute of Chemistry and Chemical Technology, Federal Research Center KSC SB RAS, 660036 Krasnoyarsk, Russia; leosol@icct.ru

[3] Institute of Chemistry, University of Tyumen, 625003 Tyumen, Russia; s.s.volkova@utmn.ru (S.S.V.); a.v.kertman@utmn.ru (A.V.K.)

[4] Department of Photonics and Laser Technology, Siberian Federal University, 660079 Krasnoyarsk, Russia; aleksandrovsky@kirensky.ru (A.S.A.)

[5] Kirensky Institute of Physics, Federal Research Center KSC SB RAS, 660036 Krasnoyarsk, Russia; dpona1@gmail.com (D.A.V.); msmolokeev@mail.ru (M.S.M.); oreshonkov@iph.krasn.ru (A.S.O.); nico@iph.krasn.ru (N.P.S.)

[6] Institute of Natural Sciences and Mathematics, Ural Federal University named after the First President of Russia B.N. Yeltsin, Mira Str. 19, 620002 Ekaterinburg, Russia; vchern@inbox.ru

[7] Institute of Physics and Technology, University of Tyumen, 625003 Tyumen, Russia; gamma125@mail.ru

[8] Research and Development Department, Kemerovo State University, Kemerovo, 650000, Russia

[9] Institute of Engineering Physics and Radioelectronic, Siberian Federal University, 660079 Krasnoyarsk, Russia

[10] School of Engineering and Construction, Siberian Federal University, Krasnoyarsk 660041, Russia;

[11] Engineering Centre of Composite Materials Based on Wolfram Compounds and Rare-Earth Elements, University of Tyumen, 625003 Tyumen, Russia; a.v.matigorov@utmn.ru

[12] Institute of Inorganic Chemistry, University of Stuttgart, D-70569 Stuttgart, Germany; thomas.schleid@iac.uni-stuttgart.de

[13] «Advanced Materials for Industry and Biomedicine» Laboratory, Kurgan State University, Sovetskaya Str. 63/4, 640020 Kurgan, Russia

[14] Innovation Center for Chemical and Pharmaceutical Technologies, Ural Federal University named after the First President of Russia B.N. Yeltsin, Mira Str. 19, 620002 Ekaterinburg, Russia

* Correspondence: a.v.rusejkina@utmn.ru (A.V.R.); damir.a.safin@gmail.com (D.A.S.)

Table S1. Structural types and space groups of the selenides $ALnCuSe_3$.[1]

La	Ce	Pr	Nd	Sm	Eu	Gd	Tb	Dy	Ho	Y	Er	Tm	Yb	Lu	Sc
EuLnCuSe₃															
Pnma											*Cmcm*				
2				2	[31]	2	2	2	2	2	[60]	2	2	2	
SrLnCuSe₃															
Pnma									*Cmcm*						
[26]	[27]	[27]	[42]	[42]		[26]	[42]	[42]	[42]	[28,42]	[42]	[42]	[42]	[26]	[36,42]
PbLnCuSe₃															
						Pnma									
						[37,39]	[37,39,40]	[37,39,40]	[37–39]	[33,39]	[37–39]	[37,39,41]	[1,37,39]	[37,39,41]	
BaLnCuSe₃															
Pnma						*Cmcm*									
[30] / [25]	[30]					[3]				[28,30]	[30]				

[1] Color code: yellow = *Pnma* space group; green = *Cmcm* space group; pink = Ba_2MnS_3 structural type [22]; blue = Eu_2CuS_3 structural type [43]; orange = $KZrCuS_3$ structural type [23]; grey = β-$BaLaCuSe_3$ structural type [30]; cyan = $BaLaCuS_3$ structural type [25].

[2] This work.

Table S2. Fractional atomic coordinates of $EuLnCuSe_3$.

Atom	*x*	*y*	*z*	Atom	*x*	*y*	*z*	Atom	*x*	*y*	*z*
	$EuLaCuSe_3$				$EuSmCuSe_3$				$EuGdCuSe_3$		
Eu	0.0916(3)	1/4	0.78634(12)	Eu	0.27628(13)	1/4	0.00032(9)	Eu	0.27194(16)	1/4	0.00035(10)
La	0.2564(3)	1/4	0.03728(11)	Sm	0.51584(12)	1/4	0.25937(10)	Gd	0.51465(16)	1/4	0.25684(14)
Cu	0.1207(5)	1/4	0.3666(3)	Cu	0.2370(3)	1/4	0.7221(2)	Cu	0.2381(4)	1/4	0.7222(2)
Se1	0.1805(4)	1/4	0.2202(2)	Se1	0.2609(2)	1/4	0.33001(14)	Se1	0.2605(2)	1/4	0.32977(15)
Se2	0.3852(4)	1/4	0.4291(2)	Se2	0.4091(2)	1/4	0.60224(16)	Se2	0.4146(2)	1/4	0.6038(2)
Se3	0.0130(4)	1/4	0.60118(19)	Se3	0.05134(18)	1/4	0.61368(15)	Se3	0.0520(2)	1/4	0.6139(2)
	$EuTbCuSe_3$				$EuDyCuSe_3$				$EuHoCuSe_3$		
Eu	0.2679(2)	1/4	0.00090(13)	Eu	0.2650(2)	1/4	0.00105(11)	Eu	0.2571(3)	1/4	0.00103(9)
Tb	0.5125(2)	1/4	0.25561(18)	Dy	0.5113(2)	1/4	0.2545(2)	Ho	0.5070(3)	1/4	0.2521(3)
Cu	0.2389(5)	1/4	0.7226(3)	Cu	0.2414(5)	1/4	0.7213(2)	Cu	0.2439(6)	1/4	0.72130(18)
Se1	0.2598(3)	1/4	0.32966(19)	Se1	0.2576(3)	1/4	0.32942(17)	Se1	0.2555(4)	1/4	0.32908(15)
Se2	0.4189(3)	1/4	0.6057(3)	Se2	0.4223(3)	1/4	0.6062(3)	Se2	0.4287(4)	1/4	0.6083(4)
Se3	0.0532(2)	1/4	0.6124(2)	Se3	0.0544(3)	1/4	0.6121(2)	Se3	0.0590(3)	1/4	0.6100(4)
	$EuYCuSe_3$				$EuTmCuSe_3$				$EuYbCuSe_3$		
Eu	0.2637(3)	1/4	0.00084(18)	Eu	0	0.75115(8)	0.25	Eu	0	0.75123(8)	1/4
Y	0.5089(4)	1/4	0.2537(4)	Tm	0	0	0	Yb	0	0	0
Cu	0.2421(7)	1/4	0.7213(3)	Cu	0	0.47090(18)	0.25	Cu	0	0.47062(18)	1/4
Se1	0.2581(4)	1/4	0.3297(2)	Se1	0	0.36007(9)	0.06277(12)	Se1	0	0.36031(10)	0.06196(11)
Se2	0.4240(4)	1/4	0.6075(4)	Se2	0	0.07871(14)	0.25	Se2	0	0.07812(15)	1/4
Se3	0.0545(4)	1/4	0.6119(4)								
	$EuLuCuSe_3$										
Eu	0	0.75130(9)	1/4								
Lu	0	0	0								
Cu	0	0.47055(19)	1/4								
Se1	0	0.36046(12)	0.06177(13)								
Se2	0	0.07798(16)	1/4								

Table S3. Anisotropic displacement parameters (Å^2) of $EuLnCuSe_3$.

Atom	U_{11}	U_{22}	U_{33}	U_{12}	U_{13}	U_{23}
			$EuLaCuSe_3$			
Eu	0.0104(18)	0.0085(16)	0.0092(16)	0.00000	0.0003(10)	0.00000
La	0.0149(16)	0.0123(18)	0.0118(16)	0.00000	−0.0034(11)	0.00000
Cu	0.019(4)	0.024(4)	0.024(4)	0.00000	0.003(3)	0.00000
Se1	0.018(3)	0.012(3)	0.013(2)	0.00000	0.0019(19)	0.00000
Se2	0.011(2)	0.014(3)	0.012(2)	0.00000	−0.0001(16)	0.00000
Se3	0.013(3)	0.011(3)	0.019(3)	0.00000	0.0025(15)	0.00000
			$EuSmCuSe_3$			
Eu	0.0241(17)	0.0127(18)	0.0155(16)	0.00000	0.0000(9)	0.00000
Sm	0.0119(16)	0.0101(17)	0.0143(16)	0.00000	0.0003(6)	0.00000
Cu	0.020(2)	0.029(3)	0.021(2)	0.00000	0.0022(17)	0.00000
Se1	0.010(2)	0.017(2)	0.0134(18)	0.00000	−0.0002(12)	0.00000
Se2	0.020(2)	0.013(2)	0.010(2)	0.00000	0.0013(11)	0.00000
Se3	0.013(2)	0.015(2)	0.012(2)	0.00000	0.0009(12)	0.00000
			$EuGdCuSe_3$			
Eu	0.0274(19)	0.0175(16)	0.0158(16)	0.00000	−0.0008(11)	0.00000
Gd	0.0119(16)	0.0129(16)	0.0175(15)	0.00000	0.0008(7)	0.00000
Cu	0.022(2)	0.022(3)	0.025(2)	0.00000	−0.001(2)	0.00000
Se1	0.017(2)	0.0174(18)	0.0096(19)	0.00000	−0.0021(16)	0.00000
Se2	0.016(2)	0.015(2)	0.017(2)	0.00000	0.0023(14)	0.00000
Se3	0.013(2)	0.018(2)	0.011(2)	0.00000	0.0007(15)	0.00000
			$EuTbCuSe_3$			
Eu	0.033(2)	0.0179(16)	0.0179(14)	0.00000	0.0029(15)	0.00000
Tb	0.0148(19)	0.0182(16)	0.0165(15)	0.00000	0.0003(10)	0.00000
Cu	0.021(3)	0.028(3)	0.022(3)	0.00000	−0.008(3)	0.00000
Se1	0.012(2)	0.0196(19)	0.0168(18)	0.00000	0.0035(19)	0.00000
Se2	0.018(2)	0.018(3)	0.019(3)	0.00000	−0.0010(19)	0.00000
Se3	0.012(2)	0.016(3)	0.017(3)	0.00000	−0.0035(18)	0.00000
			$EuDyCuSe_3$			
Eu	0.0346(18)	0.0148(13)	0.0209(12)	0.00000	0.0005(13)	0.00000
Dy	0.0121(14)	0.0117(12)	0.0172(12)	0.00000	0.0015(9)	0.00000
Cu	0.027(2)	0.021(2)	0.021(2)	0.00000	−0.003(3)	0.00000
Se1	0.0127(18)	0.0175(16)	0.0169(15)	0.00000	0.0016(19)	0.00000
Se2	0.018(2)	0.020(3)	0.020(3)	0.00000	0.0014(15)	0.00000
Se3	0.018(2)	0.012(3)	0.018(3)	0.00000	0.0001(17)	0.00000
			$EuHoCuSe_3$			
Eu	0.0379(14)	0.0118(11)	0.0187(9)	0.00000	0.000(2)	0.00000
Ho	0.0178(13)	0.0168(12)	0.0199(11)	0.00000	0.0008(9)	0.00000
Cu	0.019(2)	0.023(2)	0.025(2)	0.00000	−0.002(4)	0.00000
Se1	0.0148(15)	0.0154(15)	0.0171(13)	0.00000	0.003(3)	0.00000
Se2	0.020(2)	0.013(4)	0.021(3)	0.00000	0.000(2)	0.00000
Se3	0.016(2)	0.015(4)	0.012(3)	0.00000	−0.0032(19)	0.00000
			$EuYCuSe_3$			
Eu	0.032(2)	0.0153(16)	0.0261(15)	0.00000	−0.0015(19)	0.00000
Y	0.0144(19)	0.016(2)	0.0201(19)	0.00000	0.0000(13)	0.00000
Cu	0.027(3)	0.022(2)	0.028(3)	0.00000	−0.005(2)	0.00000
Se1	0.016(2)	0.0160(19)	0.021(2)	0.00000	−0.003(3)	0.00000
Se2	0.022(3)	0.015(3)	0.024(4)	0.00000	−0.0026(18)	0.00000
Se3	0.022(3)	0.016(3)	0.020(4)	0.00000	−0.001(2)	0.00000
			$EuTmCuSe_3$			
Eu	0.0150(12)	0.0169(12)	0.0324(15)	0.00000	0.00000	0.00000
Tm	0.0131(11)	0.0161(11)	0.0160(11)	0.00000	0.00000	0.0003(6)
Cu	0.026(2)	0.017(2)	0.0193(18)	0.00000	0.00000	0.00000
Se1	0.0153(13)	0.0138(11)	0.0168(12)	0.00000	0.00000	−0.0003(7)
Se2	0.0151(16)	0.0136(14)	0.0134(14)	0.00000	0.00000	0.00000
			$EuYbCuSe_3$			
Yb	0.0138(9)	0.0157(9)	0.0146(8)	0.00000	0.00000	0.0001(6)
Eu	0.0151(10)	0.0157(10)	0.0285(13)	0.00000	0.00000	0.00000
Cu	0.0210(18)	0.0206(18)	0.0212(17)	0.00000	0.00000	0.00000
Se1	0.0154(10)	0.0143(11)	0.0168(10)	0.00000	0.00000	−0.0004(7)

Se2	0.0147(14)	0.0155(13)	0.0127(11)	0.00000	0.00000	0.00000
			$EuLuCuSe_3$			
Eu	0.0126(10)	0.0172(9)	0.0264(10)	0.00000	0.00000	0.00000
Lu	0.0127(8)	0.0153(8)	0.0133(8)	0.00000	0.00000	0.0009(5)
Cu	0.018(2)	0.0207(18)	0.0192(16)	0.00000	0.00000	0.00000
Se1	0.0133(10)	0.0139(10)	0.0157(10)	0.00000	0.00000	0.0004(6)
Se2	0.0125(15)	0.0154(12)	0.0133(11)	0.00000	0.00000	0.00000

Table S4. Bond lengths (Å) in the structures of $EuLnCuSe_3$.

$EuLaCuSe_3$ [1]					
La–Se1	3.115(4)	Eu–Se1^{i}	2 × 3.062(3)	Cu–Se1	2.492(6)
La–Se2^{iii}	2 × 3.021(3)	Eu–Se1^{ii}	2 × 3.123(3)	Cu–Se2	2.469(6)
La–Se2^{iv}	3.193(5)	Eu–Se2^{i}	2 × 3.184(3)	Cu–Se3^{ii}	2 × 2.451(3)
La–Se3^{iii}	2 × 3.063(3)	Eu–Se3	3.156(4)		
La–Se3^{v}	3.169(4)				
$EuSmCuSe_3$ [2]					
Sm–Se1	2.902(3)	Eu–Se1^{i}	2 × 3.0965(17)	Cu–Se1^{iv}	2 × 2.5138(18)
Sm–Se1^{ii}	2.896(3)	Eu–Se2^{i}	2 × 3.174(2)	Cu–Se2	2.450(4)
Sm–Se2^{iii}	2 × 2.8836(18)	Eu–Se3^{i}	2 × 3.1585(18)	Cu–Se3	2.469(4)
Sm–Se3^{i}	2 × 2.9252(17)	Eu–Se3^{ii}	3.331(2)		
$EuGdCuSe_3$ [2]					
Gd–Se1	2.884(3)	Eu–Se1^{i}	2 × 3.089(2)	Cu–Se1^{iv}	2 × 2.505(2)
Gd–Se1^{ii}	2.869(3)	Eu–Se2^{i}	2 × 3.175(2)	Cu–Se2	2.463(4)
Gd–Se2^{iii}	2 × 2.873(3)	Eu–Se3^{i}	2 × 3.169(2)	Cu–Se3	2.461(4)
Gd–Se3^{i}	2 × 2.893(2)	Eu–Se3^{ii}	3.359(3)		
EuTbCuSe [2]					
Tb–Se1	2.857(4)	Eu–Se1^{i}	2 × 3.085(2)	Cu–Se1^{iv}	2 × 2.495 (2)
Tb–Se1^{ii}	2.860(4)	Eu–Se2^{i}	2 × 3.172(3)	Cu–Se2	2.469 (6)
Tb–Se2^{iii}	2 × 2.854(3)	Eu–Se3^{i}	2 × 3.163(3)	Cu–Se3	2.462 (5)
Tb–Se3^{i}	2 × 2.887(3)	Eu–Se3^{ii}	3.384(3)		
$EuDyCuSe_3$ [2]					
Dy–Se1	2.862(4)	Eu–Se1^{i}	2 × 3.081(2)	Cu–Se1^{iv}	2 × 2.500(2)
Dy–Se1^{ii}	2.834(4)	Eu–Se2^{i}	2 × 3.171(3)	Cu–Se2	2.455(6)
Dy–Se2^{iii}	2 × 2.851(3)	Eu–Se3^{i}	2 × 3.163(3)	Cu–Se3	2.458(5)
Dy–Se3^{i}	2 × 2.876(3)	Eu–Se3^{ii}	3.412(4)		
$EuHoCuSe_3$ [2]					
Ho–Se1	2.839(6)	Eu–Se1^{i}	2 × 3.076(2)	Cu–Se1^{iv}	2 × 2.495(2)
Ho–Se1^{ii}	2.831(6)	Eu–Se2^{i}	2 × 3.166(4)	Cu–Se2	2.463(7)
Ho–Se2^{iii}	2 × 2.845(5)	Eu–Se3^{i}	2 × 3.165(4)	Cu–Se3	2.450(6)
Ho–Se3^{i}	2 × 2.872(5)	Eu–Se3^{ii}	3.507(5)		
$EuYCuSe_3$ [2]					
Y–Se1	2.839(6)	Eu–Se1^{i}	2 × 3.078(3)	Cu–Se1^{iv}	2 × 2.504(3)
Y–Se1^{ii}	2.860(6)	Eu–Se2^{i}	2 × 3.184(4)	Cu–Se2	2.453(8)
Y–Se2^{iii}	2 x 2.850(5)	Eu–Se3^{i}	2 × 3.172(4)	Cu–Se3	2.465(8)
Y–Se3^{i}	2 x 2.868(5)	Eu–Se3^{ii}	3.424(5)		
$EuTmCuSe_3$ [3]					
Tm–Se1^{i}	4 × 2.8372(9)	Eu–Se1^{ii}	4 × 3.1725(11)	Cu–Se1	2 × 2.4547(19)
Tm–Se2	2 × 2.8162(7)	Eu–Se2^{ii}	2 × 3.0720(16)	Cu–Se2^{ii}	2 × 2.4891(17)
		Eu···Se1	2 × 3.5905(13)		
$EuYbCuSe_3$ [3]					
Yb–Se1^{i}	4 × 2.8289(9)	Eu–Se1^{ii}	4 × 3.1716(11)	Cu–Se1	2 × 2.4512(19)
Yb–Se2	2 × 2.8048(7)	Eu–Se2^{ii}	2 × 3.0749(17)	Cu–Se2^{ii}	2 × 2.4829(18)
		Eu···Se1	2 × 3.5742(13)		
$EuLuCuSe_3$ [3]					
Lu–Se1^{i}	4 × 2.8249(11)	Eu–Se1^{ii}	4 × 3.1695(13)	Cu–Se1	2 × 2.449(2)
Lu–Se2	2 × 2.8002(8)	Eu–Se2^{ii}	2 × 3.0751(19)	Cu–Se2^{ii}	2 × 2.480(2)
		Eu···Se1	2 × 3.5688(15)		

[1] Symmetry codes: (i) –*x* + 1/2, –*y*, *z* + 1/2; (ii) –*x*, *y* – 1/2, –*z* + 1; (iii) –*x* + 1/2, –*y*, *z* – 1/2; (iv) *x* – 1/2, –*y* + 1/2, –*z* + 1/2; (v) *x* + 1/2, –*y* + 1/2, –*z* + 1/2; (vi) –*x* + 1/2, –*y* + 1, *z* + 1/2; (vii) –*x*, *y* + 1/2, –*z* + 1; (viii) –*x* + 1/2, –*y* + 1, *z* – 1/2.

[2] Symmetry codes: (i) –*x* + 1/2, –*y*, *z* – 1/2; (ii) *x* + 1/2, –*y* + 1/2, –*z* + 1/2; (iii) –*x* + 1, *y* – 1/2, –*z* + 1; (iv) –*x* + 1/2, –*y*, *z* + 1/2; (v) –*x* + 1/2, –*y* + 1, *z* – 1/2; (vi) –*x* + 1, *y* + 1/2, –*z* + 1; (vii) –*x* + 1/2, –*y* + 1, *z* + 1/2.

[3] Symmetry codes: (i) –1/2 + *x*, –1/2 + *y*, *z*; (ii) –1/2 + *x*, 1/2 + *y*, *z*; (iii) 1/2 + *x*, –1/2 + *y*, *z*; (iv) –1/2 + *x*, 1/2 – *y*, –*z*; (v) –*x*, –*y*, –1/2 + *z*; (vi) 1/2 + *x*, 1/2 + *y*, *z*; (vii) –1/2 – *x*, 1/2 + *y*, 1/2 – *z*; (viii) 1/2 – *x*, 1/2 + *y*, 1/2 – *z*; (ix) –*x*, *y*, 1/2 – *z*.

Table S5. Bond angles (°) in the structures of $EuLnCuSe_3$.

		$EuLaCuSe_3$ [1]			
Se1–La–Se2^{iii}	120.13(9)	Se1^{i}–Eu–Se1^{vi}	86.95(11)	Se1–Cu–Se2	103.2(2)
Se1–La–Se2^{iv}	67.98(10)	Se1^{i}–Eu–Se1^{ii}	89.31(3)	Se1–Cu–Se3^{ii}	107.95(15)
Se1–La–Se3^{iii}	77.96(9)	Se1^{i}–Eu–Se1^{vii}	156.39(8)	Se2–Cu–Se3^{ii}	109.05(15)
Se1–La–Se3^{v}	148.64(13)	Se1^{i}–Eu–Se2^{i}	76.97(8)	Se3^{ii}–Cu–Se3^{vii}	118.5(2)
Se2^{iii}–La–Se2^{viii}	88.45(11)	Se1^{i}–Eu–Se2^{vi}	133.25(11)		
Se2^{iii}–La–Se2^{iv}	73.38(10)	Se1^{i}–Eu–Se3	77.35(9)		
Se2^{iii}–La–Se3^{iii}	88.88(7)	Se1^{ii}–Eu–Se1^{vii}	84.85(10)		
Se2^{iii}–La–Se3^{viii}	160.06(12)	Se1^{ii}–Eu–Se2^{i}	67.99(8)		
Se2^{iii}–La–Se3^{v}	80.65(9)	Se1^{ii}–Eu–Se2^{vi}	121.21(11)		
Se2^{iv}–La–Se3^{iii}	124.49(8)	Se1^{ii}–Eu–Se3	79.08(9)		
Se2^{iv}–La–Se3^{v}	143.38(11)	Se2^{i}–Eu–Se2^{vi}	82.87(10)		
Se3^{iii}–La–Se3^{viii}	86.92(11)	Se2^{i}–Eu–Se3	138.03(5)		
Se3^{iii}–La–Se3^{v}	79.41(9)				
		$EuSmCuSe_3$ [2]			
Se1–Sm–Se1^{ii}	174.62(6)	Se1^{i}–Eu–Se1^{v}	83.25(5)	Se1^{iv}–Cu–Se1^{vii}	109.82(12)
Se1–Sm–Se2^{iii}	93.19(7)	Se1^{i}–Eu–Se2^{i}	78.74(5)	Se1^{iv}–Cu–Se2	111.68(11)
Se1–Sm–Se3^{i}	89.06(6)	Se1^{i}–Eu–Se2^{v}	131.74(7)	Se1^{iv}–Cu–Se3	110.18(11)
Se1^{ii}–Sm–Se2^{iii}	90.58(6)	Se1^{i}–Eu–Se3^{i}	89.83(4)	Se2–Cu–Se3	103.13(12)
Se1^{ii}–Sm–Se3^{i}	87.12(6)	Se1^{i}–Eu–Se3^{v}	149.57(7)		
Se2^{iii}–Sm–Se2^{vi}	91.01(7)	Se1^{i}–Eu–Se3^{ii}	77.15(6)		
Se2^{iii}–Sm–Se3^{i}	89.77(4)	Se2^{i}–Eu–Se2^{v}	80.79(7)		
Se2^{iii}–Sm–Se3^{v}	177.58(8)	Se2^{i}–Eu–Se3^{i}	74.97(5)		
Se3^{i}–Sm–Se3^{v}	89.36(7)	Se2^{i}–Eu–Se3^{v}	125.79(6)		
		Se2^{i}–Eu–Se3^{ii}	139.09(4)		
		Se3^{i}–Eu–Se3^{v}	81.27(6)		
		Se3^{i}–Eu–Se3^{ii}	72.42(6)		
		$EuGdCuSe_3$ [2]			
Se1–Gd–Se1^{ii}	175.95(8)	Se1^{i}–Eu–Se1^{v}	83.09(6)	Se1^{iv}–Cu–Se1^{vii}	109.73(13)
Se1–Gd–Se2^{iii}	91.56(8)	Se1^{i}–Eu–Se2^{i}	79.92(6)	Se1^{iv}–Cu–Se2	111.49(12)
Se1–Gd–Se3^{i}	89.60(7)	Se1^{i}–Eu–Se2^{v}	132.89(9)	Se1^{iv}–Cu–Se3	110.14(12)
Se1^{ii}–Gd–Se2^{iii}	91.28(7)	Se1^{i}–Eu–Se3^{i}	89.60(5)	Se2–Cu–Se3	103.73(14)
Se1^{ii}–Gd–Se3^{i}	87.54(7)	Se1^{i}–Eu–Se3^{v}	148.25(8)		
Se2^{iii}–Gd–Se2^{vi}	90.95(11)	Se1^{i}–Eu–Se3^{ii}	76.26(6)		
Se2^{iii}–Gd–Se3^{i}	89.45(6)	Se2^{i}–Eu–Se2^{v}	80.35(8)		
Se2^{iii}–Gd–Se3^{v}	178.77(10)	Se2^{i}–Eu–Se3^{i}	75.24(6)		
Se3^{i}–Gd–Se3^{v}	90.13(9)	Se2^{i}–Eu–Se3^{v}	125.41(8)		
		Se2^{i}–Eu–Se3^{ii}	139.21(4)		
		Se3^{i}–Eu–Se3^{v}	80.54(7)		
		Se3^{i}–Eu–Se3^{ii}	71.99(7)		
		EuTbCuSe [2]			
Se1–Tb–Se1^{ii}	176.78(11)	Se1^{i}–Eu–Se1^{v}	82.91(7)	Se1^{iv}–Cu–Se1^{vii}	109.88(16)
Se1–Tb–Se2^{iii}	90.75(9)	Se1^{i}–Eu–Se2^{i}	81.00(8)	Se1^{iv}–Cu–Se2	111.11(16)
Se1–Tb–Se3^{i}	90.26(9)	Se1^{i}–Eu–Se2^{v}	134.10(11)	Se1^{iv}–Cu–Se3	110.42(16)
Se1^{ii}–Tb–Se2^{iii}	91.50(9)	Se1^{i}–Eu–Se3^{i}	88.89(7)	Se2–Cu–Se3	103.78(17)
Se1^{ii}–Tb–Se3^{i}	87.46(9)	Se1^{i}–Eu–Se3^{v}	146.67(11)		
Se2^{iii}–Tb–Se2^{vi}	91.36(12)	Se1^{i}–Eu–Se3^{ii}	75.62(8)		
Se2^{iii}–Tb–Se3^{i}	89.29(7)	Se2^{i}–Eu–Se2^{v}	80.14(9)		
Se2^{iii}–Tb–Se3^{v}	178.79(12)	Se2^{i}–Eu–Se3^{i}	75.52(7)		
Se3^{i}–Tb–Se3^{v}	90.05(11)	Se2^{i}–Eu–Se3^{v}	125.54(10)		
		Se2^{i}–Eu–Se3^{ii}	139.15(5)		
		Se3^{i}–Eu–Se3^{v}	80.44(8)		
		Se3^{i}–Eu–Se3^{ii}	71.06(9)		
		$EuDyCuSe_3$ [2]			
Se1–Dy–Se1^{ii}	177.17(12)	Se1^{i}–Eu–Se1^{v}	82.83(7)	Se1^{iv}–Cu–Se1^{vii}	109.23(14)
Se1–Dy–Se2^{iii}	90.05(10)	Se1^{i}–Eu–Se2^{i}	81.81(8)	Se1^{iv}–Cu–Se2	111.12(16)
Se1–Dy–Se3^{i}	90.36(9)	Se1^{i}–Eu–Se2^{v}	135.08(11)	Se1^{iv}–Cu–Se3	110.36(16)
Se1^{ii}–Dy–Se2^{iii}	91.93(9)	Se1^{i}–Eu–Se3^{i}	88.35(7)	Se2–Cu–Se3	104.59(16)
Se1^{ii}–Dy–Se3^{i}	87.64(9)	Se1^{i}–Eu–Se3^{v}	145.47(11)		
Se2^{iii}–Dy–Se2^{vi}	91.29(13)	Se1^{i}–Eu–Se3^{ii}	74.83(8)		
Se2^{iii}–Dy–Se3^{i}	89.22(7)	Se2^{i}–Eu–Se2^{v}	80.01(9)		

Se2^{iii}–Dy–Se3^{v}	179.34(12)	Se2^{i}–Eu–Se3^{i}	75.70(7)		
Se3^{i}–Dy–Se3^{v}	90.26(12)	Se2^{i}–Eu–Se3^{v}	125.58(10)		
		Se2^{i}–Eu–Se3^{ii}	139.15(5)		
		Se3^{i}–Eu–Se3^{v}	80.26(9)		
		Se3^{i}–Eu–Se3^{ii}	70.64(9)		
		$EuHoCuSe_3$ [2]			
Se1–Ho–Se1^{ii}	178.7(2)	Se1^{i}–Eu–Se1^{v}	82.87(6)	Se1^{iv}–Cu–Se1^{vii}	109.33(12)
Se1–Ho–Se2^{iii}	89.02(14)	Se1^{i}–Eu–Se2^{i}	83.55(11)	Se1^{iv}–Cu–Se2	110.69(19)
Se1–Ho–Se3^{i}	90.87(12)	Se1^{i}–Eu–Se2^{v}	137.60(16)	Se1^{iv}–Cu–Se3	110.71(19)
Se1^{ii}–Ho–Se2^{iii}	91.88(13)	Se1^{i}–Eu–Se3^{i}	86.88(10)	Se2–Cu–Se3	104.64(18)
Se1^{ii}–Ho–Se3^{i}	88.21(13)	Se1^{i}–Eu–Se3^{v}	142.84(16)		
Se2^{iii}–Ho–Se2^{vi}	91.4(2)	Se1^{i}–Eu–Se3^{ii}	73.84(11)		
Se2^{iii}–Ho–Se3^{i}	89.18(10)	Se2^{i}–Eu–Se2^{v}	80.02(12)		
Se2^{iii}–Ho–Se3^{v}	179.44(16)	Se2^{i}–Eu–Se3^{i}	75.79(9)		
Se3^{i}–Ho–Se3^{v}	90.27(19)	Se2^{i}–Eu–Se3^{v}	125.57(13)		
		Se2^{i}–Eu–Se3^{ii}	138.70(7)		
		Se3^{i}–Eu–Se3^{v}	80.07(12)		
		Se3^{i}–Eu–Se3^{ii}	69.00(13)		
		$EuYCuSe_3$ [2]			
Se1–Y–Se1^{ii}	178.0(2)	Se1^{i}–Eu–Se1^{v}	83.01(10)	Se1^{iv}–Cu–Se1^{vii}	109.1(2)
Se1–Y–Se2^{iii}	89.90(15)	Se1^{i}–Eu–Se2^{i}	82.14(11)	Se1^{iv}–Cu–Se2	111.2(2)
Se1–Y–Se3^{i}	91.12(14)	Se1^{i}–Eu–Se2^{v}	135.43(15)	Se1^{iv}–Cu–Se3	110.1(2)
Se1^{ii}–Y–Se2^{iii}	91.48(15)	Se1^{i}–Eu–Se3^{i}	88.20(9)	Se2–Cu–Se3	105.1(2)
Se1^{ii}–Y–Se3^{i}	87.49(14)	Se1^{i}–Eu–Se3^{v}	145.19(14)		
Se2^{iii}–Y–Se2^{vi}	91.4(2)	Se1^{i}–Eu–Se3^{ii}	74.85(11)		
Se2^{iii}–Y–Se3^{i}	88.94(10)	Se2^{i}–Eu–Se2^{v}	79.69(12)		
Se2^{iii}–Y–Se3^{v}	178.9(2)	Se2^{i}–Eu–Se3^{i}	75.78(10)		
Se3^{i}–Y–Se3^{v}	90.69(19)	Se2^{i}–Eu–Se3^{v}	125.34(14)		
		Se2^{i}–Eu–Se3^{ii}	139.20(6)		
		Se3^{i}–Eu–Se3^{v}	80.05(11)		
		Se3^{i}–Eu–Se3^{ii}	70.35(12)		
		$EuTmCuSe_3$ [3]			
Se1^{i}–Tm–Se1^{iii}	91.28(4)	Se1^{ii}–Eu–Se1^{vi}	79.50(3)	Se1–Cu–Se1^{ix}	105.67(11)
Se1^{i}–Tm–Se1^{iv}	88.71(4)	Se1^{ii}–Eu–Se1^{vii}	76.14(4)	Se1–Cu–Se2^{ii}	110.49(2)
Se1^{i}–Tm–Se2	91.85(4)	Se1^{ii}–Eu–Se1^{viii}	125.32(5)	Se2^{ii}–Cu–Se2^{vi}	109.18(11)
Se1^{i}–Tm–Se2^{v}	88.15(4)	Se1^{ii}–Eu–Se2^{ii}	85.56(3)		
		Se1^{ii}–Eu–Se2^{vi}	140.10(3)		
		Se2^{ii}–Eu–Se2^{vi}	82.65(5)		
		$EuYbCuSe_3$ [3]			
Se1^{i}–Yb–Se1^{iii}	91.39(4)	Se1^{ii}–Eu–Se1^{vi}	79.33(3)	Se1–Cu–Se1^{ix}	106.02(11)
Se1^{i}–Yb–Se1^{iv}	88.61(4)	Se1^{ii}–Eu–Se1^{vii}	76.24(4)	Se1–Cu–Se2^{ii}	110.38(2)
Se1^{i}–Yb–Se2	91.96(4)	Se1^{ii}–Eu–Se1^{viii}	125.25(6)	Se2^{ii}–Cu–Se2^{vi}	109.25(12)
Se1^{i}–Yb–Se2^{v}	88.04(4)	Se1^{ii}–Eu–Se2^{ii}	85.75(3)		
		Se1^{ii}–Eu–Se2^{vi}	140.03(3)		
		Se2^{ii}–Eu–Se2^{vi}	82.36(6)		
		$EuLuCuSe_3$ [3]			
Se1^{i}–Lu–Se1^{iii}	91.40(4)	Se1^{ii}–Eu–Se1^{vi}	79.26(4)	Se1–Cu–Se1^{ix}	106.10(12)
Se1^{i}–Lu–Se1^{iv}	88.60(4)	Se1^{ii}–Eu–Se1^{vii}	76.25(5)	Se1–Cu–Se2^{ii}	110.37(3)
Se1^{i}–Lu–Se2	92.00(4)	Se1^{ii}–Eu–Se1^{viii}	125.17(7)	Se2^{ii}–Cu–Se2^{vi}	109.22(13)
Se1^{i}–Lu–Se2^{v}	88.00(4)	Se1^{ii}–Eu–Se2^{ii}	85.85(3)		
		Se1^{ii}–Eu–Se2^{vi}	140.02(3)		
		Se2^{ii}–Eu–Se2^{vi}	82.21(6)		

[1] Symmetry codes: (i) –*x* + 1/2, –*y*, *z* + 1/2; (ii) –*x*, *y* – 1/2, –*z* + 1; (iii) –*x* + 1/2, –*y*, *z* – 1/2; (iv) *x* – 1/2, –*y* + 1/2, –*z* + 1/2; (v) *x* + 1/2, –*y* + 1/2, –*z* + 1/2; (vi) –*x* + 1/2, –*y* + 1, *z* + 1/2; (vii) –*x*, *y* + 1/2, –*z* + 1; (viii) –*x* + 1/2, –*y* + 1, *z* – 1/2.

[2] Symmetry codes: (i) –*x* + 1/2, –*y*, *z* – 1/2; (ii) *x* + 1/2, –*y* + 1/2, –*z* + 1/2; (iii) –*x* + 1, *y* – 1/2, –*z* + 1; (iv) –*x* + 1/2, –*y*, *z* + 1/2; (v) –*x* + 1/2, –*y* + 1, *z* – 1/2; (vi) –*x* + 1, *y* + 1/2, –*z* + 1; (vii) –*x* + 1/2, –*y* + 1, *z* + 1/2.

[3] Symmetry codes: (i) –1/2 + *x*, –1/2 + *y*, *z*; (ii) –1/2 + *x*, 1/2 + *y*, *z*; (iii) 1/2 + *x*, –1/2 + *y*, *z*; (iv) –1/2 + *x*, 1/2 – *y*, –*z*; (v) –*x*, –*y*, –1/2 + *z*; (vi) 1/2 + *x*, 1/2 + *y*, *z*; (vii) –1/2 – *x*, 1/2 + *y*, 1/2 – *z*; (viii) 1/2 – *x*, 1/2 + *y*, 1/2 – *z*; (ix) –*x*, *y*, 1/2 – *z*.

Table S6. The unit cell parameters for the structures of $EuLnCuSe_3$.

Compound	Space group	Structural type	*a* (Å)	*b* (Å)	*c* (Å)	*V* (Å^3)	*a*/*c*
$EuLaCuSe_3$	*Pnma*	Ba_2MnS_3	8.4389	4.2666	16.5676	596.52	0.5094
$EuCeCuSe_3$	*Pnma*	Ba_2MnS_3	8.4217	4.2574	16.4995	591.58	0.5104
$EuPrCuSe_3$	*Pnma*	Eu_2CuS_3	10.9503	4.1515	13.3802	608.27	0.8184
$EuNdCuSe_3$	*Pnma*	Eu_2CuS_3	10.8737	4.1394	13.4061	603.42	0.8111
$EuSmCuSe_3$	*Pnma*	Eu_2CuS_3	10.7522	4.1198	13.4212	594.52	0.8011
$EuGdCuSe_3$	*Pnma*	Eu_2CuS_3	10.6521	4.1012	13.4213	586.33	0.7937
$EuTbCuSe_3$	*Pnma*	Eu_2CuS_3	10.6071	4.0925	13.4172	582.43	0.7906
$EuDyCuSe_3$	*Pnma*	Eu_2CuS_3	10.5659	4.0855	13.4133	579.01	0.7877
$EuHoCuSe_3$	*Pnma*	Eu_2CuS_3	10.5309	4.0771	13.4115	575.83	0.7852
$EuYCuSe_3$	*Pnma*	Eu_2CuS_3	10.5250	4.07228	13.4029	574.46	0.7853
$EuErCuSe_3$	*Cmcm*	$KZrCuS_3$	4.0726	13.4031	10.4800	572.06	0.3886
$EuTmCuSe_3$	*Cmcm*	$KZrCuS_3$	4.0658	13.4016	10.4489	569.34	0.3891
$EuYbCuSe_3$	*Cmcm*	$KZrCuS_3$	4.0591	13.3971	10.4170	566.49	0.3897
$EuLuCuSe_3$	*Cmcm*	$KZrCuS_3$	4.0539	13.3879	10.3912	563.96	0.3901

Table S7. Bond valence calculation data for the Eu, Ln and Cu ions in the structures of $EuLnCuSe_3$.

Compound	Eu	Ln	Cu
$EuLaCuSe_3$	1.993	2.742	1.200
$EuSmCuSe_3$	1.797	3.202	1.136
$EuGdCuSe_3$	1.782	3.216	1.146
$EuTbCuSe_3$	1.792	3.167	1.155
$EuDyCuSe_3$	1.788	3.070	1.161
$EuHoCuSe_3$	1.771	3.134	1.196
$EuYCuSe_3$	1.759	3.090	1.150
$EuTmCuSe_3$	1.658	3.052	1.181
$EuYbCuSe_3$	1.655	2.966	1.196
$EuLuCuSe_3$	1.661	3.000	1.204

Table S8. Elastic constants (GPa) for $EuLnCuSe_3$.

Compound	Space group	Structural type	C_{11}	C_{12}	C_{13}	C_{22}	C_{23}	C_{33}	C_{44}	C_{55}	C_{66}
$EuLaCuSe_3$	*Pnma*	Ba_2MnS_3	108	54	62	136	46	128	27	40	46
$EuCeCuSe_3$	*Pnma*	Ba_2MnS_3	106	55	62	137	47	128	28	41	47
$EuPrCuSe_3$	*Pnma*	Eu_2CuS_3	119	36	50	139	47	84	43	22	34
$EuNdCuSe_3$	*Pnma*	Eu_2CuS_3	117	35	48	140	48	89	44	19	35
$EuSmCuSe_3$	*Pnma*	Eu_2CuS_3	118	36	47	143	49	95	46	15	36
$EuGdCuSe_3$	*Pnma*	Eu_2CuS_3	116	36	45	145	51	98	47	11	37
$EuTbCuSe_3$	*Pnma*	Eu_2CuS_3	116	37	44	145	51	101	48	11	37
$EuDyCuSe_3$	*Pnma*	Eu_2CuS_3	115	36	44	146	51	102	49	8	38
$EuHoCuSe_3$	*Pnma*	Eu_2CuS_3	116	37	44	147	51	102	49	4	38
$EuYCuSe_3$	*Pnma*	Eu_2CuS_3	117	35	47	146	50	105	49	11	38
$EuErCuSe_3$	*Cmcm*	$KZrCuS_3$	148	52	36	105	45	124	4	38	51
$EuTmCuSe_3$	*Cmcm*	$KZrCuS_3$	149	52	37	106	45	126	6	39	51
$EuYbCuSe_3$	*Cmcm*	$KZrCuS_3$	149	52	37	106	45	127	7	39	51
$EuLuCuSe_3$	*Cmcm*	$KZrCuS_3$	149	52	37	107	45	128	7	39	51

Table S9. Bulk (*B*), shear (*G*) and Young's modulus (GPa) of $EuLnCuSe_3$.

Compound	Space group	Structural type	Averaging scheme	*B*	*G*	Young's	Poisson ratio
$EuLaCuSe_3$	*Pnma*	Ba_2MnS_3	Voigt	77.3	36.6	94.9	0.295
			Reuss	77.1	34.7	90.4	0.305
			Hill	77.2	35.6	92.7	0.300
$EuCeCuSe_3$	*Pnma*	Ba_2MnS_3	Voigt	77.8	37.1	96.1	0.294
			Reuss	77.5	35.0	91.2	0.304
			Hill	77.6	36.1	93.7	0.299
$EuPrCuSe_3$	*Pnma*	Eu_2CuS_3	Voigt	67.5	33.8	86.8	0.286
			Reuss	65.7	30.3	78.9	0.300
			Hill	66.6	32.0	82.8	0.293
$EuNdCuSe_3$	*Pnma*	Eu_2CuS_3	Voigt	67.7	33.9	87.1	0.286
			Reuss	66.4	30.0	78.2	0.304
			Hill	67.1	31.9	82.7	0.295
$EuSmCuSe_3$	*Pnma*	Eu_2CuS_3	Voigt	68.8	34.2	87.9	0.287
			Reuss	67.7	28.2	74.3	0.317
			Hill	68.2	31.2	81.2	0.302
$EuGdCuSe_3$	*Pnma*	Eu_2CuS_3	Voigt	69.0	34.2	88.1	0.287
			Reuss	67.9	25.9	69.0	0.331
			Hill	68.5	30.1	78.7	0.308
$EuTbCuSe_3$	*Pnma*	Eu_2CuS_3	Voigt	69.7	34.6	89.1	0.287
			Reuss	68.5	26.0	69.2	0.332
			Hill	69.1	30.3	79.3	0.309
$EuDyCuSe_3$	*Pnma*	Eu_2CuS_3	Voigt	69.7	34.2	88.2	0.289
			Reuss	68.6	21.3	57.9	0.359
			Hill	69.1	27.8	73.4	0.323
$EuHoCuSe_3$	*Pnma*	Eu_2CuS_3	Voigt	69.8	33.8	87.3	0.292
			Reuss	68.6	14.9	41.6	0.399
			Hill	69.2	24.3	65.3	0.343
$EuYCuSe_3$	*Pnma*	Eu_2CuS_3	Voigt	70.3	35.3	90.7	0.285
			Reuss	69.6	25.5	68.3	0.337
			Hill	70.0	30.4	79.7	0.310
$EuErCuSe_3$	*Cmcm*	$KZrCuS_3$	Voigt	71.4	35.0	90.2	0.289
			Reuss	70.4	15.3	42.8	0.399
			Hill	70.9	25.1	67.4	0.341
$EuTmCuSe_3$	*Cmcm*	$KZrCuS_3$	Voigt	71.8	35.5	91.5	0.288
			Reuss	70.9	18.4	50.7	0.381
			Hill	71.4	26.9	71.8	0.332
$EuYbCuSe_3$	*Cmcm*	$KZrCuS_3$	Voigt	72.2	35.9	92.3	0.287
			Reuss	71.3	20.3	55.7	0.370
			Hill	71.7	28.1	74.5	0.327
$EuLuCuSe_3$	*Cmcm*	$KZrCuS_3$	Voigt	72.5	36.2	93.0	0.286
			Reuss	71.6	21.4	58.5	0.364
			Hill	72.0	28.8	76.2	0.324

Table S10. Calculated IR wavenumbers (cm^{-1}) for $EuLnCuSe_3$.

Pnma space group																				*Cmcm* space group							
Ba_2MnS_3 structural type				Eu_2CuS_3 structural type																$KZrCuS_3$ structural type							
$EuLaCuSe_3$		$EuCeCuSe_3$		$EuPrCuSe_3$		$EuNdCuSe_3$		$EuSmCuSe_3$		$EuGdCuSe_3$		$EuTbCuSe_3$		$EuDyCuSe_3$		$EuHoCuSe_3$		$EuYCuSe_3$		$EuErCuSe_3$		$EuTmCuSe_3$		$EuYbCuSe_3$		$EuLuCuSe_3$	
B_{1u}	45.9	B_{1u}	46.0	B_{3u}	52.9	B_{3u}	52.3	B_{3u}	50.8	B_{3u}	49.9	B_{3u}	49.4	B_{3u}	48.8	B_{3u}	48.9	B_{3u}	52.2	B_{1u}	45.8	B_{1u}	46.3	B_{1u}	46.3	B_{1u}	46.5
B_{2u}	59.9	B_{2u}	60.6	B_{1u}	62.1	B_{1u}	61.6	B_{1u}	60.9	B_{1u}	60.2	B_{1u}	59.7	B_{1u}	59.2	B_{1u}	59.1	B_{1u}	58.0	B_{2u}	82.0	B_{2u}	81.3	B_{2u}	80.3	B_{2u}	79.8
B_{3u}	61.9	B_{3u}	62.2	B_{3u}	75.3	B_{3u}	75.5	B_{3u}	76.1	B_{3u}	76.2	B_{3u}	76.0	B_{3u}	75.7	B_{3u}	75.6	B_{3u}	74.6	B_{1u}	91.3	B_{1u}	90.7	B_{1u}	89.7	B_{1u}	89.0
B_{3u}	79.8	B_{3u}	80.1	B_{1u}	83.3	B_{1u}	83.8	B_{1u}	84.3	B_{2u}	82.5	B_{2u}	83.3	B_{2u}	82.7	B_{2u}	84.5	B_{1u}	83.9	B_{1u}	93.9	B_{1u}	94.2	B_{1u}	94.2	B_{1u}	94.6
B_{3u}	90.5	B_{3u}	90.4	B_{2u}	87.3	B_{2u}	84.6	B_{2u}	86.5	B_{1u}	84.6	B_{1u}	84.8	B_{1u}	85.0	B_{1u}	85.0	B_{2u}	90.6	B_{2u}	100.4	B_{2u}	99.8	B_{2u}	99.5	B_{2u}	99.3
B_{1u}	90.6	B_{1u}	90.9	B_{3u}	93.3	B_{3u}	93.4	B_{3u}	93.4	B_{3u}	92.7	B_{3u}	92.5	B_{3u}	91.9	B_{3u}	91.5	B_{2u}	98.7	B_{3u}	100.5	B_{3u}	100.5	B_{3u}	100.8	B_{3u}	100.4
B_{3u}	92.6	B_{3u}	92.2	B_{2u}	96.3	B_{2u}	93.9	B_{3u}	95.5	B_{3u}	95.3	B_{3u}	95.4	B_{3u}	95.0	B_{3u}	95.5	B_{3u}	99.3	B_{3u}	109.5	B_{3u}	108.9	B_{3u}	108.0	B_{3u}	107.5
B_{2u}	97.3	B_{2u}	97.1	B_{3u}	96.5	B_{3u}	96.4	B_{1u}	99.8	B_{2u}	95.7	B_{2u}	97.6	B_{2u}	98.4	B_{1u}	101.1	B_{1u}	101.0	B_{3u}	132.2	B_{3u}	131.1	B_{3u}	129.3	B_{3u}	126.9
B_{1u}	103.2	B_{1u}	102.1	B_{1u}	99.4	B_{1u}	99.4	B_{2u}	101.2	B_{1u}	100.2	B_{1u}	100.3	B_{1u}	100.5	B_{2u}	102.9	B_{3u}	101.7	B_{2u}	148.1	B_{2u}	149.0	B_{2u}	147.8	B_{2u}	147.6
B_{1u}	108.3	B_{1u}	108.9	B_{3u}	104.5	B_{3u}	104.3	B_{3u}	104.8	B_{3u}	105.1	B_{3u}	105.4	B_{3u}	105.7	B_{3u}	105.9	B_{3u}	108.3	B_{1u}	154.3	B_{1u}	153.7	B_{1u}	152.3	B_{1u}	151.6
B_{3u}	115.6	B_{3u}	115.3	B_{1u}	110.8	B_{1u}	111.3	B_{1u}	111.2	B_{1u}	110.9	B_{1u}	110.6	B_{1u}	110.1	B_{1u}	109.9	B_{1u}	117.6	B_{3u}	175.0	B_{3u}	175.3	B_{3u}	175.8	B_{3u}	176.2
B_{1u}	125.6	B_{1u}	125.6	B_{1u}	135.3	B_{1u}	135.4	B_{1u}	133.1	B_{1u}	131.9	B_{1u}	131.8	B_{1u}	130.2	B_{1u}	129.7	B_{2u}	143.7	B_{1u}	181.3	B_{1u}	180.6	B_{1u}	179.1	B_{1u}	178.5
B_{1u}	133.6	B_{1u}	134.0	B_{2u}	141.2	B_{2u}	141.3	B_{2u}	147.0	B_{2u}	141.6	B_{2u}	142.6	B_{2u}	146.0	B_{2u}	143.7	B_{1u}	153.3	B_{3u}	191.6	B_{3u}	190.2	B_{3u}	188.4	B_{3u}	187.5
B_{3u}	135.5	B_{3u}	135.3	B_{1u}	155.1	B_{1u}	155.5	B_{3u}	156.0	B_{3u}	155.5	B_{2u}	152.0	B_{3u}	154.6	B_{2u}	151.6	B_{1u}	158.0	B_{2u}	196.4	B_{1u}	197.0	B_{1u}	197.1	B_{1u}	197.6
B_{2u}	139.1	B_{2u}	140.4	B_{2u}	157.6	B_{2u}	156.1	B_{1u}	156.0	B_{1u}	156.3	B_{3u}	155.4	B_{1u}	156.2	B_{3u}	154.1	B_{3u}	169.8	B_{1u}	196.5	B_{2u}	197.2	B_{2u}	198.7	B_{2u}	199.4
B_{3u}	145.3	B_{3u}	146.4	B_{3u}	157.7	B_{3u}	157.5	B_{2u}	159.6	B_{2u}	158.2	B_{1u}	156.3	B_{2u}	158.1	B_{1u}	155.9	B_{2u}	170.1	B_{3u}	206.2	B_{3u}	206.0	B_{3u}	205.8	B_{3u}	206.1
B_{2u}	155.4	B_{2u}	156.0	B_{1u}	169.3	B_{2u}	169.4	B_{1u}	171.6	B_{1u}	172.9	B_{1u}	173.5	B_{1u}	174.0	B_{1u}	174.3	B_{1u}	175.0								
B_{1u}	159.6	B_{1u}	159.9	B_{2u}	174.2	B_{1u}	170.0	B_{1u}	178.2	B_{1u}	179.3	B_{1u}	179.5	B_{3u}	180.0	B_{3u}	179.4	B_{1u}	181.1								
B_{3u}	167.5	B_{1u}	166.8	B_{1u}	176.2	B_{1u}	176.8	B_{3u}	179.7	B_{3u}	180.4	B_{3u}	180.5	B_{1u}	180.1	B_{1u}	180.5	B_{3u}	182.3								
B_{1u}	167.6	B_{3u}	167.2	B_{3u}	177.2	B_{3u}	178.5	B_{3u}	180.5	B_{3u}	181.4	B_{3u}	181.8	B_{3u}	182.0	B_{3u}	182.3	B_{3u}	187.2								
B_{3u}	174.6	B_{3u}	174.7	B_{3u}	177.9	B_{3u}	178.8	B_{3u}	185.0	B_{2u}	183.0	B_{3u}	186.8	B_{3u}	187.3	B_{3u}	187.6	B_{1u}	192.9								
B_{1u}	181.5	B_{1u}	181.7	B_{3u}	184.8	B_{3u}	185.0	B_{2u}	186.0	B_{3u}	186.3	B_{2u}	188.4	B_{2u}	190.9	B_{1u}	192.3	B_{3u}	193.7								
B_{1u}	186.7	B_{1u}	186.5	B_{3u}	191.9	B_{3u}	192.7	B_{1u}	193.3	B_{1u}	193.3	B_{1u}	193.5	B_{1u}	192.8	B_{2u}	195.1	B_{2u}	195.0								
B_{3u}	197.8	B_{3u}	197.8	B_{1u}	192.4	B_{1u}	192.8	B_{3u}	194.2	B_{3u}	195.4	B_{3u}	195.9	B_{3u}	196.2	B_{3u}	196.6	B_{3u}	198.2								
B_{2u}	207.4	B_{2u}	207.0	B_{1u}	196.8	B_{1u}	197.9	B_{1u}	198.7	B_{1u}	198.7	B_{1u}	198.3	B_{1u}	197.2	B_{1u}	196.8	B_{3u}	211.7								
B_{1u}	208.2	B_{1u}	208.4	B_{3u}	200.6	B_{3u}	200.5	B_{3u}	199.9	B_{3u}	199.3	B_{3u}	198.9	B_{3u}	198.1	B_{3u}	197.8	B_{1u}	218.3								
B_{3u}	223.5	B_{3u}	223.2	B_{1u}	211.3	B_{1u}	210.5	B_{1u}	207.3	B_{1u}	206.7	B_{1u}	206.7	B_{1u}	206.2	B_{1u}	206.3	B_{1u}	228.7								

Table S11. Calculated Raman wavenumbers (cm^{-1}) for $EuLnCuSe_3$.

Pnma space group																				*Cmcm* space group							
Ba_2MnS_3 structural type				Eu_2CuS_3 structural type																$KZrCuS_3$ structural type							
$EuLaCuSe_3$		$EuCeCuSe_3$		$EuPrCuSe_3$		$EuNdCuSe_3$		$EuSmCuSe_3$		$EuGdCuSe_3$		$EuTbCuSe_3$		$EuDyCuSe_3$		$EuHoCuSe_3$		$EuYCuSe_3$		$EuErCuSe_3$		$EuTmCuSe_3$		$EuYbCuSe_3$		$EuLuCuSe_3$	
B_{1g}	41.2	B_{1g}	41.9	A_g	29.9	A_g	27.6	A_g	22.9	A_g	20.4	A_g	18.9	A_g	17.4	A_g	18.2	15.9	A_g	B_{2g}	62.2	B_{2g}	63.4	B_{1g}	64.1	B_{1g}	63.9
A_g	42.8	A_g	43.1	A_g	40.1	A_g	41.2	A_g	42.8	A_g	44.5	A_g	45.3	A_g	45.8	A_g	46.4	52.8	A_g	B_{1g}	64.2	B_{1g}	64.1	B_{2g}	64.3	B_{2g}	65.5
A_g	63.9	A_g	64.4	B_{1g}	52.3	B_{1g}	68.7	B_{1g}	50.6	B_{1g}	50.7	B_{1g}	51.2	B_{1g}	50.6	B_{1g}	50.8	58.6	B_{1g}	A_g	67.7	A_g	67.5	A_g	67.4	A_g	67.3
B_{3g}	66.9	B_{3g}	67.7	B_{2g}	60.0	B_{3g}	96.4	B_{2g}	61.1	B_{3g}	60.0	B_{2g}	61.3	B_{3g}	61.3	B_{2g}	61.4	62.6	B_{3g}	B_{2g}	96.1	B_{2g}	95.6	B_{2g}	95.1	B_{2g}	94.6
B_{1g}	67.2	B_{1g}	67.9	B_{3g}	60.9	B_{2g}	99.9	B_{3g}	62.0	B_{2g}	61.2	B_{3g}	61.4	B_{2g}	61.3	B_{3g}	62.5	63.4	B_{2g}	B_{1g}	101.0	B_{1g}	100.3	B_{1g}	100.0	B_{1g}	99.6
B_{2g}	69.1	B_{2g}	69.2	B_{1g}	65.8	B_{1g}	112.2	B_{3g}	68.3	B_{1g}	64.0	B_{1g}	65.9	B_{1g}	66.3	B_{2g}	67.6	65.6	B_{1g}	A_g	105.5	A_g	105.1	A_g	105.0	A_g	104.9
B_{2g}	75.7	B_{2g}	76.0	A_g	68.5	B_{3g}	159.0	B_{2g}	68.4	B_{3g}	66.3	B_{3g}	66.9	B_{3g}	66.6	B_{3g}	67.8	66.2	B_{2g}	B_{3g}	146.1	B_{3g}	146.7	B_{3g}	146.1	B_{3g}	145.6
A_g	81.0	A_g	80.8	B_{3g}	69.2	A_g	167.0	B_{1g}	68.9	B_{2g}	67.9	B_{2g}	67.4	B_{2g}	66.9	A_g	68.4	66.8	A_g	B_{1g}	150.0	B_{1g}	150.5	B_{1g}	149.9	B_{1g}	149.4
B_{2g}	82.0	B_{2g}	82.7	B_{2g}	70.8	B_{2g}	180.4	A_g	69.1	A_g	69.0	A_g	68.8	A_g	68.6	B_{1g}	69.0	78.1	B_{3g}	B_{2g}	159.0	B_{2g}	158.7	B_{2g}	158.1	B_{2g}	157.7
B_{3g}	85.0	B_{3g}	86.0	B_{2g}	79.1	B_{2g}	184.3	B_{2g}	77.6	B_{2g}	76.8	B_{2g}	76.8	B_{2g}	76.9	B_{2g}	77.1	86.0	B_{2g}	A_g	173.3	A_g	173.6	A_g	173.7	A_g	174.0
A_g	85.8	A_g	86.4	A_g	96.9	B_{3g}	195.0	A_g	96.4	A_g	96.0	A_g	96.3	A_g	96.4	B_{2g}	96.5	96.0	B_{2g}	A_g	185.0	A_g	185.0	A_g	185.0	A_g	185.1
B_{3g}	101.0	B_{3g}	101.1	B_{3g}	97.2	A_g	210.4	B_{2g}	96.8	B_{2g}	96.4	B_{2g}	96.6	B_{2g}	96.4	A_g	96.9	97.3	B_{3g}	B_{2g}	193.3	B_{2g}	193.5	B_{2g}	193.3	B_{2g}	193.3
B_{1g}	102.5	B_{1g}	102.6	B_{2g}	97.9	B_{2g}	60.4	A_g	101.2	B_{3g}	96.6	B_{3g}	98.3	B_{3g}	99.0	A_g	102.7	102.4	A_g	B_{1g}	195.1	B_{1g}	195.8	A_g	196.2	A_g	196.3
B_{2g}	104.7	B_{2g}	104.7	A_g	99.1	B_{1g}	68.2	B_{3g}	102.1	B_{1g}	98.8	B_{1g}	100.2	B_{1g}	100.4	B_{3g}	103.5	104.20	B_{1g}	A_g	196.3	A_g	196.2	B_{1g}	197.3	B_{1g}	198.0
A_g	118.0	A_g	118.4	B_{1g}	101.6	A_g	88.4	B_{1g}	104.2	A_g	102.2	A_g	102.8	A_g	102.7	B_{1g}	104.6	104.23	A_g	B_{2g}	198.9	B_{2g}	199.3	B_{2g}	199.4	B_{2g}	199.7
B_{2g}	122.9	B_{2g}	122.6	B_{2g}	106.9	B_{2g}	143.3	B_{2g}	108.2	B_{2g}	109.3	A_g	109.1	A_g	108.5	A_g	108.3	113.6	A_g								
B_{3g}	126.1	A_g	127.1	A_g	112.3	A_g	154.3	A_g	110.4	A_g	109.6	B_{2g}	109.5	B_{2g}	109.4	B_{2g}	109.4	114.2	B_{2g}								
B_{2g}	127.7	B_{3g}	127.2	B_{1g}	144.7	B_{1g}	169.8	B_{2g}	144.0	B_{2g}	142.9	B_{2g}	142.7	B_{2g}	141.3	B_{2g}	140.9	148.0	B_{1g}								
A_g	127.8	B_{2g}	127.5	B_{2g}	145.2	B_{2g}	51.7	B_{1g}	150.8	B_{1g}	145.6	B_{1g}	146.9	B_{1g}	150.3	B_{1g}	148.2	152.0	B_{3g}								
B_{1g}	140.1	B_{1g}	141.5	B_{3g}	147.1	B_{3g}	64.0	B_{3g}	152.3	B_{3g}	146.8	B_{3g}	148.8	B_{3g}	151.9	B_{3g}	151.0	156.5	B_{2g}								
B_{2g}	142.6	B_{2g}	142.5	B_{2g}	157.8	B_{1g}	98.4	A_g	157.6	A_g	157.2	B_{3g}	153.5	A_g	156.5	B_{3g}	152.4	162.1	B_{2g}								
A_g	145.5	A_g	145.8	B_{3g}	158.5	B_{3g}	144.8	B_{2g}	158.7	B_{2g}	159.1	B_{1g}	154.1	B_{2g}	158.9	B_{1g}	154.1	169.3	B_{3g}								
A_g	149.0	A_g	149.4	B_{1g}	159.1	B_{2g}	157.3	B_{3g}	161.1	B_{1g}	160.5	A_g	157.2	B_{3g}	160.2	A_g	156.1	171.5	A_g								
B_{2g}	156.5	B_{2g}	156.4	A_g	159.3	A_g	170.9	B_{1g}	161.6	B_{3g}	160.5	B_{2g}	159.0	B_{1g}	160.5	B_{2g}	158.8	171.6	B_{1g}								
B_{1g}	161.1	B_{3g}	162.3	A_g	165.8	A_g	0.0	A_g	169.1	A_g	170.8	A_g	171.6	A_g	172.0	A_g	172.6	172.0	A_g								
B_{3g}	161.1	B_{1g}	162.3	B_{2g}	172.4	B_{3g}	61.6	B_{2g}	174.6	B_{2g}	176.0	B_{2g}	176.6	B_{2g}	176.9	B_{2g}	177.2	179.6	B_{2g}								
A_g	164.2	A_g	164.1	B_{3g}	173.2	B_{1g}	83.8	A_g	181.7	B_{3g}	181.9	A_g	182.7	A_g	181.9	A_g	181.6	184.7	A_g								
B_{2g}	169.5	B_{2g}	169.8	B_{1g}	175.5	B_{2g}	99.4	A_g	183.3	A_g	182.3	A_g	184.2	A_g	184.4	A_g	184.7	189.0	B_{2g}								
A_g	174.6	A_g	175.0	A_g	179.6	A_g	111.3	B_{3g}	185.0	A_g	183.7	B_{3g}	187.2	B_{2g}	186.4	B_{2g}	186.4	193.7	B_{3g}								
B_{2g}	185.2	B_{2g}	185.6	A_g	184.3	A_g	135.4	B_{1g}	187.0	B_{1g}	183.9	B_{2g}	187.2	B_{3g}	189.6	B_{2g}	192.5	194.9	A_g								
A_g	189.2	A_g	189.8	B_{2g}	189.9	B_{2g}	155.5	B_{2g}	188.2	B_{2g}	187.4	B_{1g}	189.3	B_{1g}	191.9	B_{3g}	194.0	195.8	B_{1g}								
B_{2g}	197.8	B_{2g}	198.3	B_{2g}	191.9	B_{2g}	170.0	B_{2g}	192.3	B_{2g}	192.4	B_{2g}	192.7	B_{2g}	192.7	B_{1g}	195.9	197.7	A_g								
A_g	208.7	A_g	208.8	B_{2g}	193.4	B_{2g}	176.8	A_g	196.1	A_g	197.1	A_g	197.2	A_g	197.1	A_g	197.0	199.1	B_{2g}								
B_{3g}	211.7	B_{3g}	211.7	A_g	194.3	A_g	192.8	B_{2g}	196.5	B_{2g}	197.6	B_{2g}	197.8	B_{2g}	198.2	B_{2g}	198.7	213.4	B_{2g}								
B_{1g}	212.0	B_{1g}	212.0	A_g	210.0	A_g	197.9	A_g	210.2	A_g	210.5	A_g	210.4	A_g	210.2	A_g	210.0	226.4	A_g								
B_{2g}	226.1	B_{2g}	226.3	B_{2g}	226.2	B_{2g}	210.5	B_{2g}	225.7	B_{2g}	226.3	B_{2g}	226.4	B_{2g}	225.6	B_{2g}	225.3	246.8	B_{2g}								

Table S12. Calculated wavenumbers (cm^{-1}) of "silent" modes (type of modes is A_u) for $EuLnCuSe_3$.

***Pnma* space group**										**_Cmcm_ space group**			
Ba_2MnS_3 structural type		Eu_2CuS_3 structural type								$KZrCuS_3$ structural type			
$EuLaCuSe_3$	$EuCeCuSe_3$	$EuPrCuSe_3$	$EuNdCuSe_3$	$EuSmCuSe_3$	$EuGdCuSe_3$	$EuTbCuSe_3$	$EuDyCuSe_3$	$EuHoCuSe_3$	$EuYCuSe_3$	$EuErCuSe_3$	$EuTmCuSe_3$	$EuYbCuSe_3$	$EuLuCuSe_3$
39.5	40.0	60.9	60.4	61.1	60.1	60.7	60.4	60.6	65.9	60.5	60.2	59.4	59.3
85.2	86.2	69.3	68.2	70.5	66.4	68.0	68.2	69.9	73.6	144.2	145.2	144.1	144.0
97.4	97.2	91.2	88.4	95.6	91.4	92.7	93.4	97.8	92.3				
125.8	126.8	143.9	143.3	148.8	142.8	144.9	147.7	146.9	148.3				
154.6	155.3	155.7	154.3	158.7	157.6	150.6	157.1	149.5	167.1				
207.2	206.8	174.4	169.8	185.6	182.4	187.7	190.2	194.4	194.3				

Table S13. Calculated IR wavenumbers (cm^{-1}) and mode intensities ($km\ mol^{-1}$) for $EuLnCuSe_3$ (Ln = La, Tb, Y and Tm).

***Pnma* space group**									***Cmcm* space group**		
Ba_2MnS_3 structural type			Eu_2CuS_3 structural type						$KZrCuS_3$ structural type		
$EuLaCuSe_3$			$EuTbCuSe_3$			$EuYCuSe_3$			$EuTmCuSe_3$		
Mode	Wavenumber	Intensity	Mode	Wavenumber	Intensity	Mode	Wavenumber	Intensity	Mode	Wavenumber	Intensity
B_{1u}	45.9	0	B_{3u}	49.4	28.75	B_{3u}	52.2	55	B_{1u}	46.3	19.47
B_{2u}	59.9	4.03	B_{1u}	59.7	1.47	B_{1u}	58.0	0.64	B_{2u}	81.3	5.38
B_{3u}	61.9	5.02	B_{3u}	76.0	0.89	B_{3u}	74.6	0.88	B_{1u}	90.7	4.1
B_{3u}	79.8	5.91	B_{2u}	83.3	9.68	B_{1u}	83.9	3.48	B_{1u}	94.2	118.11
B_{3u}	90.5	0.01	B_{1u}	84.8	5.08	B_{2u}	90.6	6.29	B_{2u}	99.8	20.79
B_{1u}	90.6	215.62	B_{3u}	92.5	70.56	B_{2u}	98.7	34.25	B_{3u}	100.5	144.36
B_{3u}	92.6	22.14	B_{3u}	95.4	185.92	B_{3u}	99.3	0.02	B_{3u}	108.9	80.6
B_{2u}	97.3	48.26	B_{2u}	97.6	35	B_{1u}	101.0	366.34	B_{3u}	131.1	2.11
B_{1u}	103.2	3.38	B_{1u}	100.3	288.5	B_{3u}	101.7	228.64	B_{2u}	149.0	632.71
B_{1u}	108.3	16.26	B_{3u}	105.4	2.48	B_{3u}	108.3	3.88	B_{1u}	153.7	477.11
B_{3u}	115.6	58.76	B_{1u}	110.6	167.51	B_{1u}	117.6	161.75	B_{3u}	175.3	62.24
B_{1u}	125.6	136.94	B_{1u}	131.8	6.15	B_{2u}	143.7	8.64	B_{1u}	180.6	81.56
B_{1u}	133.6	25.96	B_{2u}	142.6	133.03	B_{1u}	153.3	0.00	B_{3u}	190.2	207.14
B_{3u}	135.5	947.69	B_{2u}	152.0	1224.28	B_{1u}	158.0	0.46	B_{1u}	197.0	23.15
B_{2u}	139.1	198.42	B_{3u}	155.4	980.84	B_{3u}	169.8	985.96	B_{2u}	197.2	64.19
B_{3u}	145.3	185.9	B_{1u}	156.3	0	B_{2u}	170.1	1534.75	B_{3u}	206.0	33.88
B_{2u}	155.4	885.36	B_{1u}	173.5	122.11	B_{1u}	175.0	55.72			
B_{1u}	159.6	500.57	B_{1u}	179.5	36.56	B_{1u}	181.1	2.19			
B_{3u}	167.5	326.8	B_{3u}	180.5	136.57	B_{3u}	182.3	15.50			
B_{1u}	167.6	17.32	B_{3u}	181.8	12.84	B_{3u}	187.2	0.24			
B_{3u}	174.6	48.62	B_{3u}	186.8	45.73	B_{1u}	192.9	2.54			
B_{1u}	181.5	685.56	B_{2u}	188.4	150.83	B_{3u}	193.7	89.40			
B_{1u}	186.7	9.36	B_{1u}	193.5	169.57	B_{2u}	195.0	189.98			
B_{3u}	197.8	57.61	B_{3u}	195.9	34.1	B_{3u}	198.2	343.61			
B_{2u}	207.4	397.55	B_{1u}	198.3	261.03	B_{3u}	211.7	120.11			
B_{1u}	208.2	87.86	B_{3u}	198.9	25.82	B_{1u}	218.3	484.18			
B_{3u}	223.5	4.92	B_{1u}	206.7	23.38	B_{1u}	228.7	89.37			

Table S14. Calculated Raman wavenumbers (cm^{-1}) and mode intensities (a.u.) for $EuLnCuSe_3$ (Ln = La, Tb, Y and Tm).

***Pnma* space group**						***Cmcm* space group**		
Ba_2MnS_3 structural type			Eu_2CuS_3 structural type			$KZrCuS_3$ structural type		
$EuLaCuSe_3$			$EuTbCuSe_3$			$EuTmCuSe_3$		
Mode	Wavenumber	Intensity	Mode	Wavenumber	Intensity	Mode	Wavenumber	Intensity
B_{1g}	41.2	315	A_g	18.9	627	B_{2g}	63.4	736
A_g	42.8	147	A_g	45.3	54	B_{1g}	64.1	535
A_g	63.9	264	B_{1g}	51.2	10	A_g	67.5	853
B_{3g}	66.9	716	B_{2g}	61.3	2	B_{2g}	95.6	313
B_{1g}	67.2	6	B_{3g}	61.4	322	B_{1g}	100.3	211
B_{2g}	69.1	210	B_{1g}	65.9	5	A_g	105.1	251
B_{2g}	75.7	231	B_{3g}	66.9	396	B_{3g}	146.7	93
A_g	81.0	575	B_{2g}	67.4	887	B_{1g}	150.5	42
B_{2g}	82.0	2	A_g	68.8	917	B_{2g}	158.7	9
B_{3g}	85.0	139	B_{2g}	76.8	2	A_g	173.6	174
A_g	85.8	161	A_g	96.3	13	A_g	185.0	124
B_{3g}	101.0	41	B_{2g}	96.6	341	B_{2g}	193.5	169
B_{1g}	102.5	1000	B_{3g}	98.3	178	B_{1g}	195.8	0
B_{2g}	104.7	48	B_{1g}	100.2	22	A_g	196.2	1000
A_g	118.0	259	A_g	102.8	186	B_{2g}	199.3	159
B_{2g}	122.9	19	A_g	109.1	132			
B_{3g}	126.1	9	B_{2g}	109.5	1			
B_{2g}	127.7	76	B_{2g}	142.7	3			
A_g	127.8	860	B_{1g}	146.9	93			
B_{1g}	140.1	8	B_{3g}	148.8	7			
B_{2g}	142.6	5	B_{3g}	153.5	21			
A_g	145.5	74	B_{1g}	154.1	28			
A_g	149.0	320	A_g	157.2	16			
B_{2g}	156.5	0	B_{2g}	159.0	18			
B_{1g}	161.1	75	A_g	171.6	304			
B_{3g}	161.1	653	B_{2g}	176.6	4			
A_g	164.2	922	A_g	182.7	83			
B_{2g}	169.5	68	A_g	184.2	141			
A_g	174.6	826	B_{3g}	187.2	1			
B_{2g}	185.2	25	B_{2g}	187.2	4			
A_g	189.2	389	B_{1g}	189.3	0			
B_{2g}	197.8	30	B_{2g}	192.7	234			
A_g	208.7	373	A_g	197.2	1000			
B_{3g}	211.7	115	B_{2g}	197.8	104			
B_{1g}	212.0	66	A_g	210.4	89			
B_{2g}	226.1	30	B_{2g}	226.4	2			

Table S15. Wavenumbers (cm^{-1}) and types of the phonon modes at the Γ-point for $EuTbCuSe_3$ and $EuTmCuSe_3$.

Pnma **space group**

Eu_2CuS_3 structural type

$EuTbCuSe_3$

Wavenumber	Mode	IR [1]	Raman [1]	Involved ions [2,3]
18.9	A_g	I	A	Eu^S, Tb^S, Cu, Se1^S, Se2^S, Se3
45.3	A_g	I	A	Eu, Tb^S, Cu, Se1, Se2, Se3^S
49.4	B_{3u}	A	I	Eu^S, Tb^S, Cu, Se1, Se2, Se3
50.5	B_{1g}	I	A	Eu^W, Tb^S, Cu, Se1, Se2, Se3
59.7	B_{1u}	A	I	Eu, Tb, Cu, Se2, Se3
60.8	A_u	I	I	Eu^W, Tb^S, Cu^W, Se1, Se2, Se3^W
61.3	B_{2g}	I	A	Eu^S, Cu^W, Se1^W, Se2^W
62.5	B_{3g}	I	A	Eu^S, Tb, Cu^W, Se1^W, Se2^W, Se3
67.4	B_{2g}	I	A	Eu^S, Cu^S, Se3
67.8	B_{3g}	I	A	Eu, Tb^S, Cu^W, Se1, Se2^W, Se3^W
68.8	A_g	I	A	Eu^S, Tb, Cu, Se2, Se3
69.5	B_{1g}	I	A	Eu^S, Cu^W, Se1, Se2
70.4	A_u	I	I	Eu^S, Cu^S, Se1^W, Se2^W, Se3
76.0	B_{3u}	A	I	Eu, Cu^S, Se1, Se2, Se3
76.7	B_{2g}	I	A	Tb, Cu, Se1, Se2, Se3
84.8	B_{1u}	A	I	Eu, Tb, Cu, Se1^W, Se2^W, Se3
85.6	B_{2u}	A	I	Eu^W, CuS, Se1, Se2S
92.5	B_{3u}	A	I	Eu, Cu^S, Se1^W, Se3
95.4	B_{3u}	A	I	Tb, Cu^S, Se1, Se2, Se3^W
96.3	A_g	I	A	Eu, Tb, Cu^W, Se1, Se2
96.6	B_{2g}	I	A	Eu, Cu^S, Se3
97.8	A_u	I	I	Eu^W, Tb, Cu^S, Se1, Se2, Se3^W
100.4	B_{1u}	A	I	Eu^W, Cu^S, Se1, Se2
102.5	A_g	I	A	Eu, Cu^S, Se3
103.2	B_{2u}	A	I	Eu, Tb, Cu^S, Se3
104.0	B_{3g}	I	A	Eu, Tb^W, Cu^S, Se1, Se2^W, Se3^W
105.2	B_{1g}	I	A	Eu, Tb^W, Cu, Se1^W, Se2, Se3^W
105.4	B_{3u}	A	I	Eu, Cu, Se1^W, Se2
109.1	A_g	I	A	Eu^W, Tb^W, Cu^S, Se1, Se2
109.6	B_{2g}	I	A	Eu, Tb^W, Cu, Se1, Se2^W
110.6	B_{1u}	A	I	Eu, Tb, Cu, Se1^W, Se2^W
131.8	B_{1u}	A	I	Tb, Cu, Se1^W, Se3^W
142.7	B_{2g}	I	A	Tb^W, Se1^S, Se2^W
146.3	B_{2u}	A	I	Tb^W,Se1^S
150.0	A_u	I	I	Eu^W, Tb, Cu^W, Se1^W, Se2^W
150.3	B_{1g}	I	A	Tb^W, Se1^S, Se2^W
151.8	A_u	I	I	Tb^W, Se1^S
153.0	B_{3g}	I	A	Tb, Cu, Se1, Se2, Se3^W
154.4	B_{2u}	A	I	Cu^W, Se1, Se2, Se3
155.1	B_{3g}	I	A	Tb^W, Cu, Se1, Se2, Se3^W
155.4	B_{3u}	A	I	Tb^W, Se2^S
156.3	B_{1u}	A	I	Tb^W, Se2^S
156.9	B_{1g}	I	A	Cu^W, Se1, Se2, Se3
157.2	A_g	I	A	Tb^W, Se2^S
159.1	B_{2g}	I	A	Tb^W, Se2^S
171.5	A_g	I	A	Se1, Se2, Se3
173.5	B_{1u}	A	I	Se1, Se2^W, Se3
176.6	B_{2g}	I	A	Tb^W, Se1^W, Se2^W, Se3
179.5	B_{1u}	A	I	Cu, Se1, Se2^W, Se3
180.6	B_{3u}	A	I	Tb^W, Cu, Se1^W, Se2, Se3
181.8	B_{3u}	A	I	Cu^W, Se1, Se2, Se3
182.7	A_g	I	A	Cu, Se3^S
184.1	A_g	I	A	Tb^W, Cu^W, Se1^W, Se2^W, Se3
186.9	B_{3u}	A	I	Cu, Se3^S
187.2	B_{2g}	I	A	Cu, Se2, Se3^S
192.7	B_{2g}	I	A	Cu, Se1^W, Se2^W, Se3
193.5	B_{1u}	A	I	Cu, Se3^S

Cmcm **space group**

$KZrCuS_3$ structural type

$EuTmCuSe_3$

Wavenumber	Mode	IR [1]	Raman [1]	Involved ions [2,4]
46.3	B_{1u}	A	I	Eu^S, Tm^S, Cu, Se1^S, Se2
60.1	A_u	I	I	Tm^S, Se1^S
64.0	B_{2g}	I	A	Eu^S, Cu, Se1
64.1	B_{1g}	I	A	Eu^S, Cu^S, Se1, Se2^S
67.5	A_g	I	A	Eu^S, Cu^S, Se1, Se2^S
81.3	B_{2u}	A	I	Eu^S, Tm^S, Cu^S, Se1, Se2
90.6	B_{1u}	A	I	Eu, Tm^S, Cu^S, Se1^S, Se2
94.2	B_{1u}	A	I	Eu, Tm, Cu^S,Se1, Se2^W
95.6	B_{2g}	I	A	Eu, Cu^S, Se1^S
99.8	B_{2u}	A	I	Eu^S, Cu^S, Se2
100.3	B_{1g}	I	A	Eu^S, Cu^S, Se1^W, Se2^S
100.6	B_{3u}	A	I	Eu^S, Tm^W, Cu^S, Se1, Se2
105.1	A_g	I	A	Eu^S, Cu^S, Se1, Se2
108.8	B_{3u}	A	I	Eu^S, Tm, Cu, Se1
131.2	B_{3u}	A	I	Tm^S, Cu, Se1^W, Se2^W
143.9	A_u	I	I	Tm, Se1^S
145.6	B_{3g}	I	A	Se1^S
147.7	B_{2u}	A	I	Eu^W, Tm, Se1^S
149.5	B_{1g}	I	A	Eu^W, Se1^S
153.7	B_{1u}	A	I	Tm, Cu, Se1, Se2
158.7	B_{2g}	I	A	Eu^W, Cu, Se1^S, Se2
173.6	A_g	I	A	Eu^W, Se1^S, Se2
175.3	B_{3u}	A	I	Se1, Se2^S
180.6	B_{1u}	A	I	Tm, Cu^W, Se1, Se2^S
185.0	A_g	I	A	Cu^S, Se1^W, Se2^S
190.2	B_{3u}	A	I	Tm, Cu, Se1^S
193.5	B_{2g}	I	A	Cu, Se1, Se2^S
195.8	B_{1g}	I	A	Cu^S, Se2^S
196.2	A_g	I	A	Cu, Se1^S, Se2^W
197.0	B_{1u}	A	I	Tm^W, Cu^S, Se1, Se2
197.1	B_{2u}	A	I	Cu^S, Se2^S
199.3	B_{2g}	I	A	Cu^S, Se1, Se2^W
206.0	B_{3u}	A	I	Tm^W, Cu^S, Se1, Se2

194.3	B_{3g}	I	A	Cu, Se1^{W}, Se3
194.7	A_{u}	I	I	Tb^{W}, Cu, Se1^{W}, Se2
195.3	B_{2u}	A	I	Cu^{W}, Se2^{W}, Se3^{S}
195.9	B_{3u}	A	I	Tb^{W}, Cu^{W}, Se1, Se3
196.1	B_{1g}	I	A	Eu^{W}, Cu^{W}, Se1, Se2^{W}, Se3
197.1	A_{g}	I	A	Cu, Se1, Se2, Se3^{W}
197.8	B_{2g}	I	A	Cu, Se1, Se2, Se3^{W}
198.3	B_{1u}	A	I	Tb^{W}, Cu, Se2, Se3^{W}
198.9	B_{3u}	A	I	Cu, Se1^{W}, Se2
206.7	B_{1u}	A	I	Tb^{W}, Cu, Se1, Se2^{W}, Se3^{W}
210.4	A_{g}	I	A	Tb^{W}, Cu, Se1^{W}, Se2, Se3^{W}
226.3	B_{2g}	I	A	Tb^{W}, Cu, Se1^{W}, Se2, Se3^{W}

[1] A = active mode, I = inactive mode.

[2] Superscripts "S" and "W" denote strong and weak ion displacements in the mode, respectively. If the displacement is 0.02–0.03 Å, it is denoted as "S"; if the displacement is 0.005–0.01 Å, it is denoted as "W"; if the displacement is <0.005 Å, the ion is omitted from consideration.

[3] The maximum displacement of 0.03 Å is for the Eu ion in the mode at 18.9 cm^{-1}.

[4] The maximum displacement of 0.04 Å is for the Eu and Cu ions in the modes at 64.0 and 95.6 cm^{-1}, respectively.

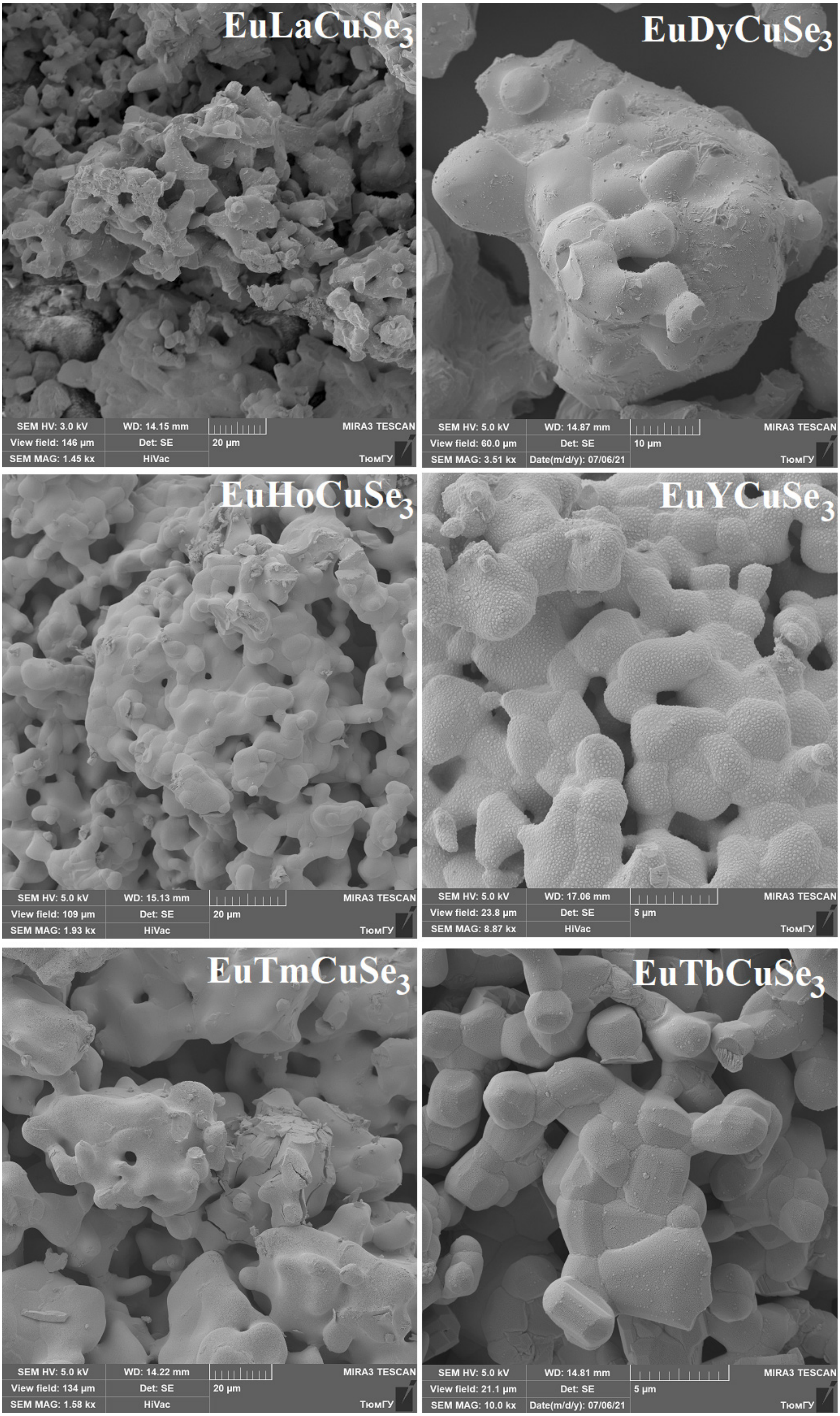

Figure S1. SEM images of $EuLnCuSe_3$.

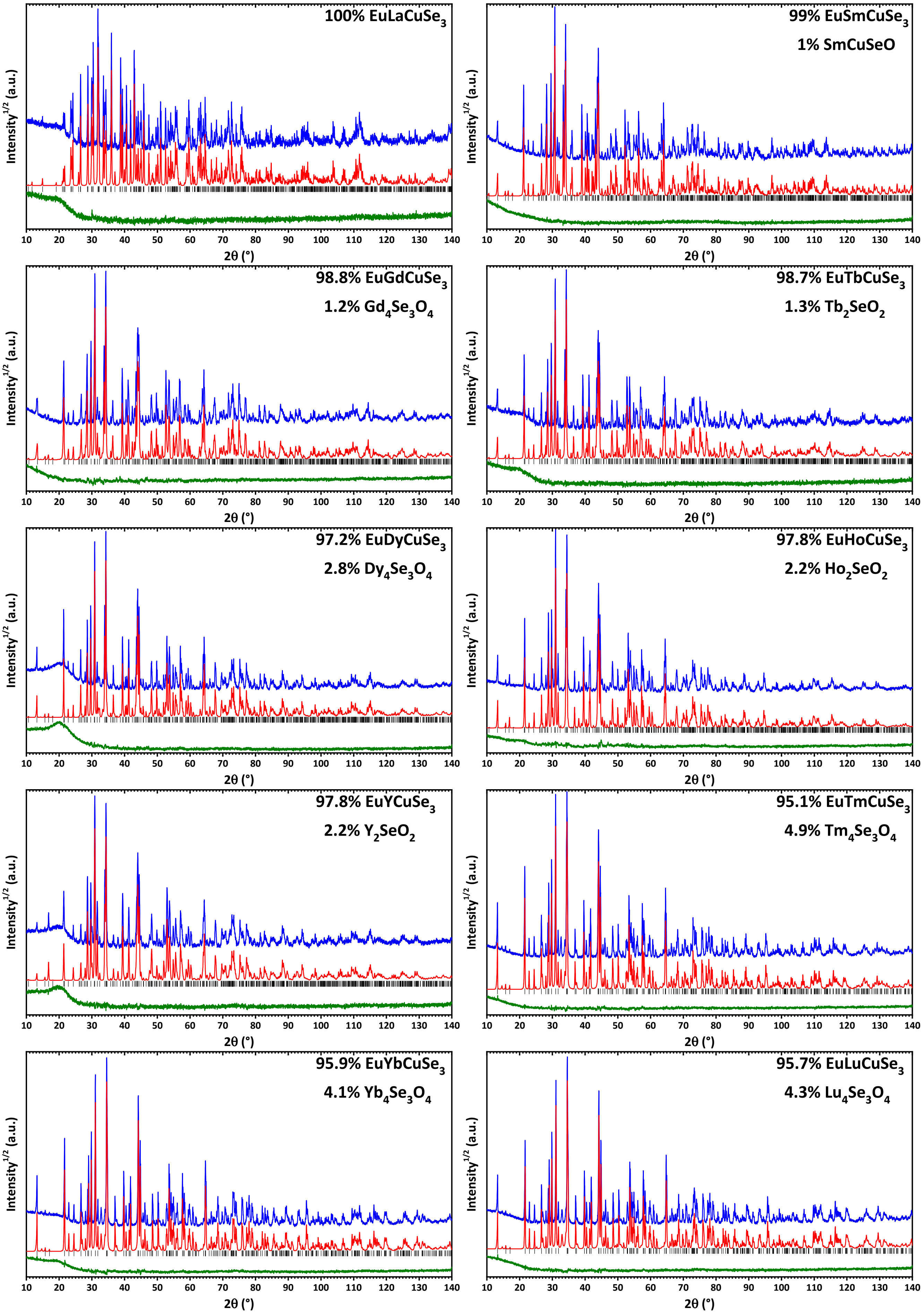

Figure S2. Observed (blue), calculated (red) and difference (green) X-ray powder diffraction patterns for $EuLnCuSe_3$ after crystal structure refinement.

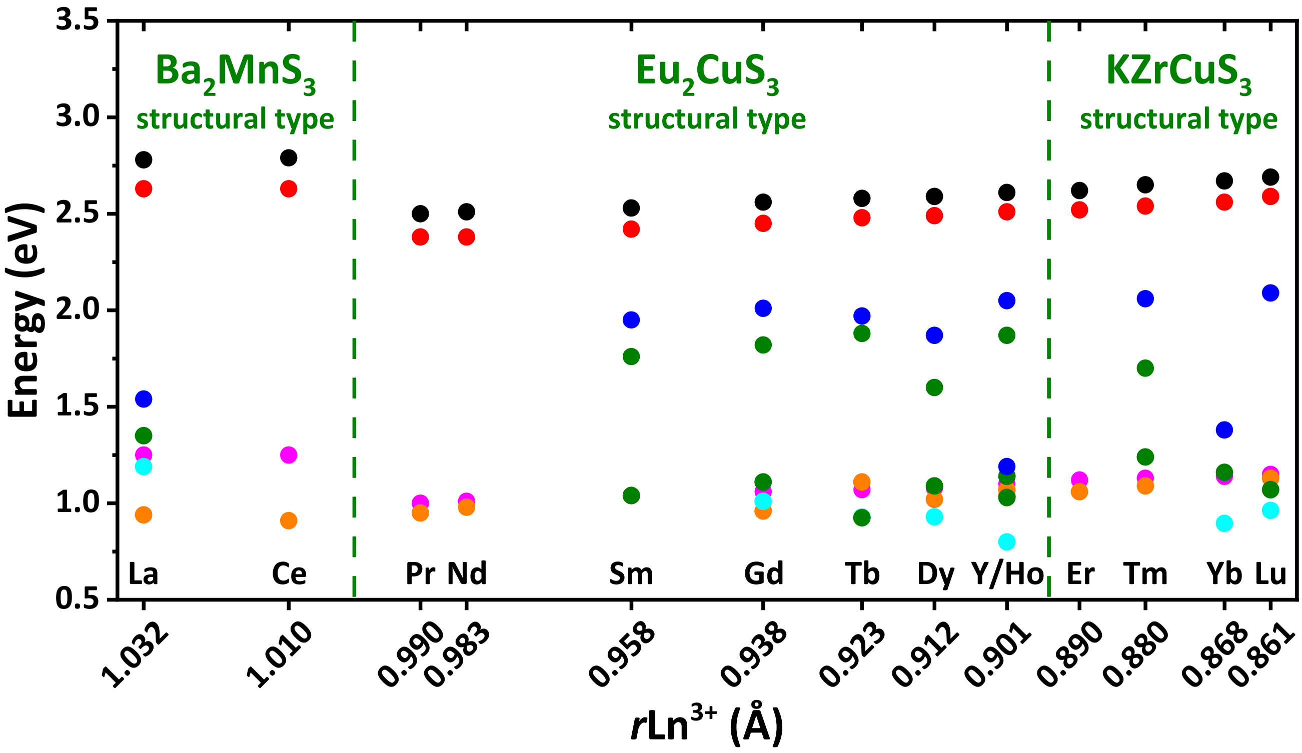

Figure S3. The band gap values of EuLnCuSe3. Black = band gap from the PBE0 simulation (this work, Table 2); red = band gap from the B3LYP simulation (this work, Table 2); blue = experimental direct band gap (this work, Table 2); green = band gap from uncorrected Kubelka-Munk function; magenta = PBE simulation (this work, Table 2); cyan = experimental indirect band gap (this work, Table 2); orange = non-hybrid PBE simulation [32].

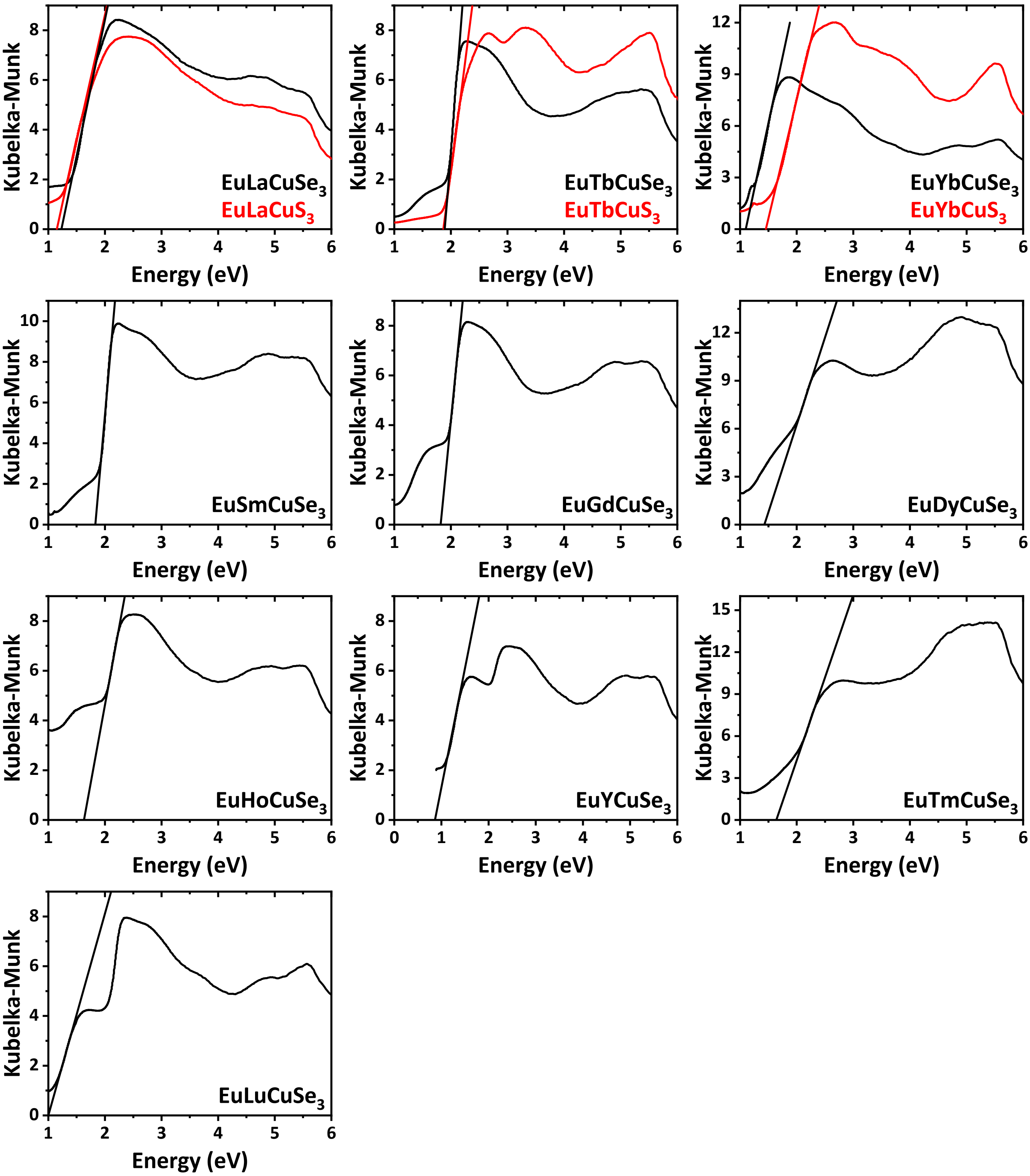

Figure S4. The Kubelka-Munk spectra of $EuLnCuSe_3$, and $EuLaCuS_3$, $EuTbCuS_3$ and $EuYbCuS_3$.

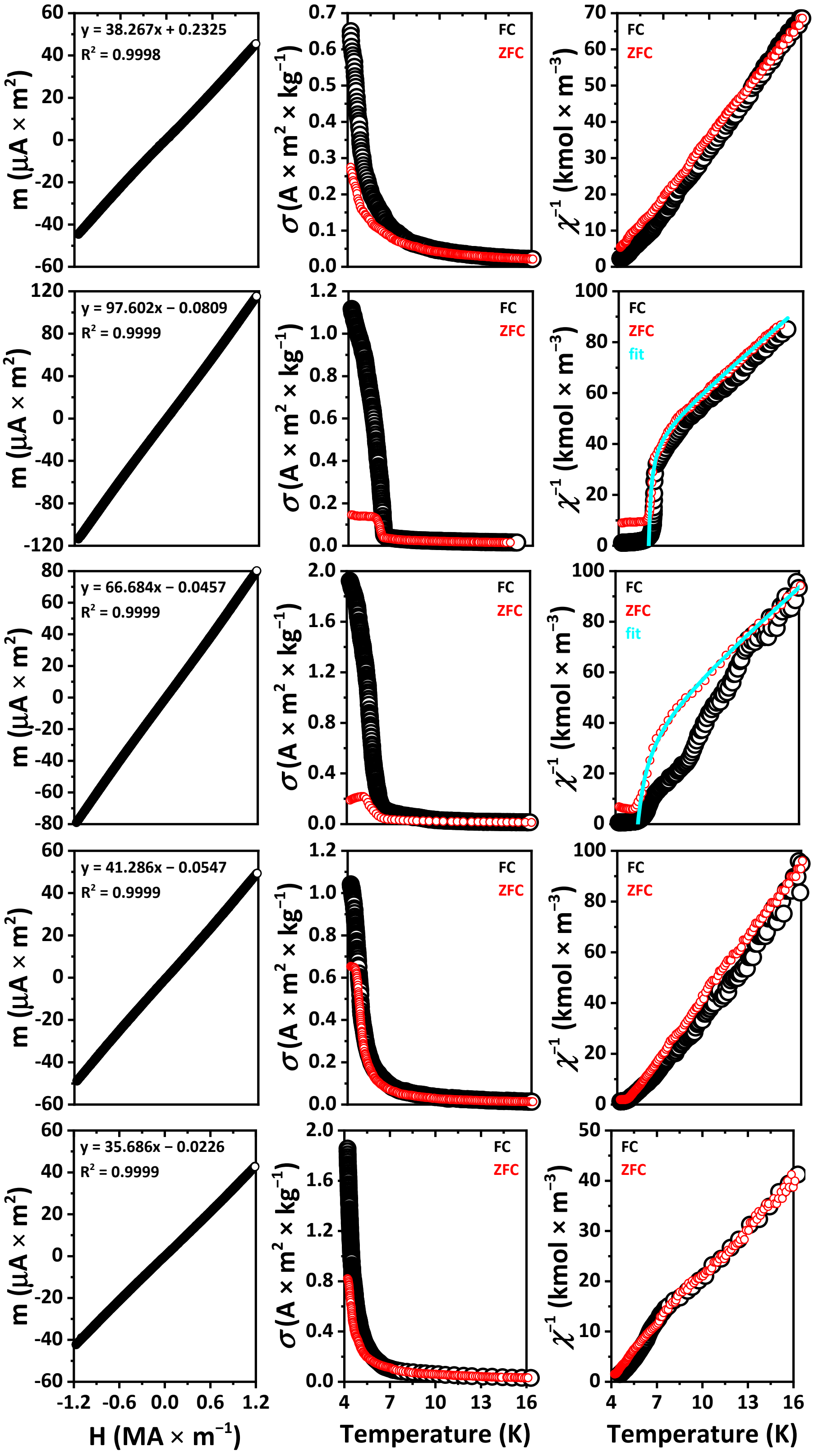

Figure S5. Field-dependent magnetic moments at 296 K (left), and temperature-dependent specific magnetization (middle) and reciprocal magnetic susceptibility (right) of (from top to bottom) $EuYCuSe_3$, $EuTbCuSe_3$, $EuDyCuSe_3$, $EuYbCuSe_3$ and $EuLuCuSe_3$, respectively, at 10 Oe. The measurements of low-temperature magnetization were performed in the zero-field cooled (ZFC) and nonzero-field cooled (FC) modes. Fit line shows approximation by the Néel model of a ferrimagnet [80].
